# Clinical characteristics, molecular epidemiology and mechanisms of colistin heteroresistance in *Enterobacter cloacae* complex

**DOI:** 10.3389/fcimb.2025.1536058

**Published:** 2025-03-06

**Authors:** Chunli Wei, Jiming Wu, Jisheng Zhang, Youtao Liang, Kaixin Yu, Mingjing Liao, Xushan Liang, Jianmin Wang, Wenzhang Long, Jin Wang, Shijian Chen, Yang Yang, Xue Gong, Jie Li, Xiaoli Zhang

**Affiliations:** ^1^ Department of Microbiology, Yongchuan Hospital of Chongqing Medical University, Chongqing, China; ^2^ Department of Pathogenic Biology, Basic Medicine of Jiamusi University, Jiamusi, China

**Keywords:** *Enterobacter cloacae* complex, colistin heteroresistance, resistant subpopulations, prevalence, risk factors, mechanisms

## Abstract

**Introduction:**

Colistin has emerged as the last resort for treating multidrug-resistant *Enterobacter cloacae* complex (ECC) infections. The primary purposes of this study were to demonstrate the presence of colistin heteroresistance in ECC and to further investigate their clinical characteristics, molecular epidemiology and mechanisms.

**Methods:**

Population analysis profiles (PAP) were performed to confirm the heteroresistance phenotype. Average nucleotide identity (ANI) was determined to classify ECC species. Phylogenetic analysis based on core genome single nucleotide polymorphisms (cg-SNPs), multilocus sequence typing (MLST) and core genome MLST (cg-MLST). Risk factors and clinical outcomes of infections were analyzed through a retrospective case-control study. Potential mechanisms of colistin heteroresistance were evaluated using polymerase chain reaction (PCR), efflux pump inhibition assays and reverse transcription quantitative PCR (RT-qPCR).

**Results:**

A high proportion (24.4%) of the non-resistant strains were colistin-heteroresistant isolates. Among the several ECC species, *Enterobacter kobei* had the largest percentage (29.4%) of colistin-heteroresistant isolates, followed by *Enterobacter hormaechei* (20.5%) and *Enterobacter bugandensis* (20.0%). Notably, only one strain (0.8%; 1/132) of *Enterobacter hormaechei* was fully resistant to colistin. Different ECC species showed varying heteroresistance levels: *Enterobacter roggenkampii*, *Enterobacter kobei*, *Enterobacter asburiae* and *Enterobacter bugandensis* displayed high heteroresistance levels  (MIC ≥ 128 mg/L). 75% of all ST116 and ST56 strains were heteroresistant to colistin. The infection of ST116 and ST56 strains as well as exposure to cephalosporin antibiotics were independent risk factors for colistin-heteroresistant ECC infections. Mechanistic analysis revealed that heteroresistance strongly correlated with the overexpression of *arnA*, regulated by the PhoPQ two-component system (TCS). Notably, *mgrB* had minimal impact. AcrAB-TolC efflux pump genes showed unsynchronized expression; High *acrB* expression was strongly associated with colistin heteroresistance, while *acrA* and *tolC* were not.

**Discussion:**

Colistin heteroresistance showed species-dependent variations in levels and prevalence rates. The colistin-heteroresistant mechanisms were complex, involving coordinated regulation of multiple genes. These results highlighted the need for tailored antimicrobial stewardship. In addition, the development of direct, reliable and rapid clinical methods for detecting heteroresistance is essential for improving infection management and prevention.

## 1 Introduction

*Enterobacter cloacae* complex (ECC) has emerged as one of the leading causes of nosocomial infections worldwide (Sanders Jr. and Sanders, 1997). This pathogen is inherently resistant to first- and second-generation cephalosporins due to the overexpression of an inducible AmpC β-lactamase, along with increasing resistance to third-generation cephalosporins, aminoglycosides and fluoroquinolones (Liu et al., 2021b). Although the carbapenem antimicrobials with broad-spectrum resistance are seen as an essential choice for the treatment of infections with these multidrug-resistant pathogens, ECC has become the third most prevalent species within Enterobacteriaceae associated with hospital-acquired infections (Zhou et al., 2018; Liu et al., 2021b). Ceftazidime/avibactam (CAZ/AVI) is a widely used novel β-lactam/β-lactamase inhibitor combination in China and it has shown good clinical efficacy against carbapenem-resistant Enterobacteriaceae (Qu et al., 2023). However, avibactam does not inhibit metallo-β-lactamases (MBLs). As a result, CAZ/AVI cannot be used to treat patients infected with MBL-producing ECC isolates. Because of these circumstances, colistin is now a last-resort therapy choice for managing these life-threatening infections (Ortwine et al., 2015; Ezadi et al., 2019; Nang et al., 2021). Colistin primarily exerts its effects by binding to phospholipids in the bacterial cell membrane, disrupting its structural integrity and ultimately resulting in bacterial lysis and death (Falagas and Kasiakou, 2005). However, the overuse and improper application of colistin have led to the emergence of colistin-heteroresistant ECC (Napier et al., 2014). Clinical characteristics, molecular epidemiology and mechanisms of colistin heteroresistance in ECC were still not sufficiently understood.

Colistin heteroresistance has been observed in various Gram-negative pathogens and factors such as pathogen type, geographical region and detection methods have a significant impact on its prevalence. A retrospective study in the United States showed that *Enterobacter* spp. had the highest rate of colistin heteroresistance (21.6%), followed by *Klebsiella* spp. (8.4%) and *Escherichia* (2.1%) (Band et al., 2021). Similarly, 27.5% (38/138) of the ECC strains from a university hospital in Japan were identified as colistin-heteroresistant isolates (Fukuzawa et al., 2023). In contrast, a multicenter study in China found the colistin heteroresistance rate of carbapenem-resistant *Klebsiella pneumoniae* was 6.2% (Weng et al., 2023). These studies demonstrated the prevalence of colistin heteroresistance in *Enterobacter* spp., especially in ECC, was significantly higher than that observed in other members of the Enterobacteriaceae family. Traditional drug susceptibility testing methods often mistakenly classified heteroresistant isolates as susceptible strains, which posed a major challenge to clinical treatment. Previous studies indicated that the *arnBCADTEF* operon mediated the 4-amino-4-deoxy-L-arabinose (L-Ara4N) modification of lipid A, which was regulated by the PhoPQ two-component regulatory system (TCS). The TCS was negatively controlled by *mgrB* (Kang et al., 2019; Doijad et al., 2023). The *ecr* gene may activate the *arnBCADTEF* operon through the PhoPQ TCS, thereby conferring colistin heteroresistance in ECC (Huang et al., 2019). The *dedA* gene which encodes an inner membrane protein, was also essential for colistin heteroresistance in ECC (Huang et al., 2019). Colistin heteroresistance in ECC was associated with the overexpression of AcrAB-tolC efflux pump regulated by *soxRS* (Telke et al., 2017). However, the diversity and complexity of ECC species classification complicated our understanding of clinical characteristics, epidemiology and mechanisms within colistin-heteroresistant ECC. Consequently, accurate ECC species identification is crucial for effective diagnosis and treatment.

ECC represents a highly diverse bacterial group, including 12 species and 22 evolutionary lineages (Leister and Hugler, 2022; Huang et al., 2023). Average nucleotide identity (ANI) analysis based on whole-genome sequencing (WGS) offered a method with superior accuracy and resolution for ECC species identification (Fukuzawa et al., 2023). In this study, we investigated the diversity of ECC species in this region by ANI analysis, concentrating on their heteroresistance and full resistance to colistin. We also retrospectively evaluated the risk factors and clinical outcomes associated with colistin-heteroresistant ECC infections and preliminarily explored the potential mechanisms for colistin heteroresistance in ECC. Our research aims to provide valuable theoretical insights and practical references for improving clinical treatment.

## 2 Materials and methods

### 2.1 Clinical strains and antimicrobial susceptibility testing

In this study, 212 non-repetitive clinical ECC strains were collected and monoclonal purified from September 2018 to July 2023 in a teaching hospital. Colistin susceptibility testing was performed using the broth microdilution method, with *Escherichia coli* ATCC 25922 used as a quality control strain. The results were interpreted according to the breakpoints established by the European Committee on Antimicrobial Susceptibility Testing (http://www.eucast.org/clinical_breakpoints/).

### 2.2 Initial screening for colistin heteroresistance

An initial screening for colistin heteroresistance was conducted on colistin-sensitive isolates or those presenting a skip well phenomenon in multiple tests. The activated isolates were adjusted to the McFarland 0.5 turbidity standard and inoculated onto Mueller-Hinton agar plates. Subsequently, the antibiotic disk containing 10 μg of colistin (Liofilchem, Italy) and the E-test strip (BIO-KONT, China) were quickly placed on these plates and incubated at 37°C for 24 hours. A strain was deemed heteroresistant to colistin if visible colony growth occurs within the circle of inhibition on the antibiotic disk or E-test strip (Hung et al., 2012).

### 2.3 Population analysis profile analysis and bacterial passage testing

The PAP confirmation analysis was performed on colistin-heteroresistant isolates obtained from the initial screening. The activated isolates were adjusted to the McFarland 0.5 turbidity standard and serially diluted in a 10-fold gradient until they reached a final concentration of 1.5 × 10^3^ CFU/mL. 100 µL aliquots from each concentration of strain dilutions were spread onto plates containing varying concentrations of colistin (0, 0.5, 1, 2, 4, 6, 8, 16 mg/L) and incubated at 37°C for 48 hours. Colony counts were recorded as the average value from three independent experiments. Colistin heteroresistance was defined as the detection of resistant subpopulations present at high frequencies (greater than 1 × 10^-7^) and with MICs at least eightfold higher than clinically susceptible parental populations. The colonies on the plates with the highest colistin concentrations represented resistant subpopulations preserved for subsequent experiments. These resistant subpopulations were activated and mixed in antibiotic-free Luria-Bertani broth at a ratio of 1:1000 and then incubated at 37°C with shaking for 20 hours, corresponding to 10 generations. This process was repeated until resistant subpopulations reached 50 generations. The stability of the resistant subpopulations was assessed by determining their change in the MIC compared to its initial MIC before passaging.

### 2.4 Whole-genome sequencing and analysis

WGS was performed on the HiSeq PE150 platform (Illumina). Sequence assembly was conducted using SPAdes (v3.15.5) (Bankevich et al., 2012). Resistance gene sequences were identified using the Resistance Gene Identifier model from the Comprehensive Antibiotic Resistance Database (Alcock et al., 2020). Multilocus sequence typing (MLST) was analyzed utilizing the PubMLST tools (https://pubmlst.org/). The corresponding core genome MLST (cg-MLST) was performed using the chewBBACA method (Silva et al., 2018). FastANI was employed to rapidly calculate ANI and classify ECC species (Jain et al., 2018). Core genome single nucleotide polymorphisms (cg-SNPs) was performed using bwa, samtools and GATK software (Li and Durbin, 2009; Li et al., 2009; Van der Auwera et al., 2013).

### 2.5 Clinical information collection

A retrospective case-control study was conducted to analyze host-related risk factors and clinical outcomes in patients infected with colistin-heteroresistant isolates. Detailed patient characteristics were collected from the medical records system and microbiology database, including age, gender, department of admission, infection sources, underlying diseases and conditions, invasive procedures within the past month, antimicrobial exposure within the past three months and other parameters related to clinical outcomes.

### 2.6 Detection of antibiotic resistance genes and efflux pump inhibition assays

Polymerase chain reaction (PCR) was performed with the primers specified in **Supplementary Table S1**. The resulting products were sent to Sangon Biotech (China) for Sanger sequencing to analyze *mgrB*, *phoP* and *phoQ* gene mutations. Efflux pump inhibitors (EPIs) carbonyl cyanide m-chlorophenylhydrazone (CCCP, 10 mg/L, Sigma) and Phe-Arg-β-naphthylamide (PAβN, 25 mg/L, Sigma) were used to assess the efflux pump activity in colistin-heteroresistant isolates. The impact of EPIs on colistin heteroresistance was evaluated by measuring the MIC of colistin in the presence and absence of CCCP and PAβN. A reduction of 4-fold or greater in MIC upon adding EPIs indicated a significant inhibitory effect (Zhong et al., 2014).

### 2.7 Quantitative expression of colistin-heteroresistance-related genes

To evaluate the expression of colistin resistance genes (*mgrB*, *phoP*, *phoQ*, *arnA*), RND efflux pump genes (*acrA*, *acrB*, *tolC*) and their global transcriptional regulators (*ramA*, *soxS*), six colistin-heteroresistant parental strains and their resistant subpopulations were selected for reverse transcription quantitative PCR (RT-qPCR). Primers were supplied in **Supplementary Table S1**. Three technical replicates for each sample and all experiments were performed in triplicate.

### 2.8 Statistical analysis

Categorical variables were compared using Fisher’s exact test or chi-square test, with results presented as frequencies and percentages. Continuous variables were calculated using t-tests or Wilcoxon’s rank sum test, depending on whether the data were normally distributed and the median and interquartile range (IQR) were reported. Variables having a p-value < 0.05 from the univariate analysis were included as potential covariates in the multivariate logistic regression model. Odds ratios (OR) and 95% confidence intervals (CI) were calculated. The p-value of < 0.05 was considered statistically significant. All statistical analyses were performed using SPSS 25.0 (Chicago, Illinois).

## 3 Results

### 3.1 The discovery of colistin-heteroresistant isolates and general characteristics of all strains

**Figure 1:**
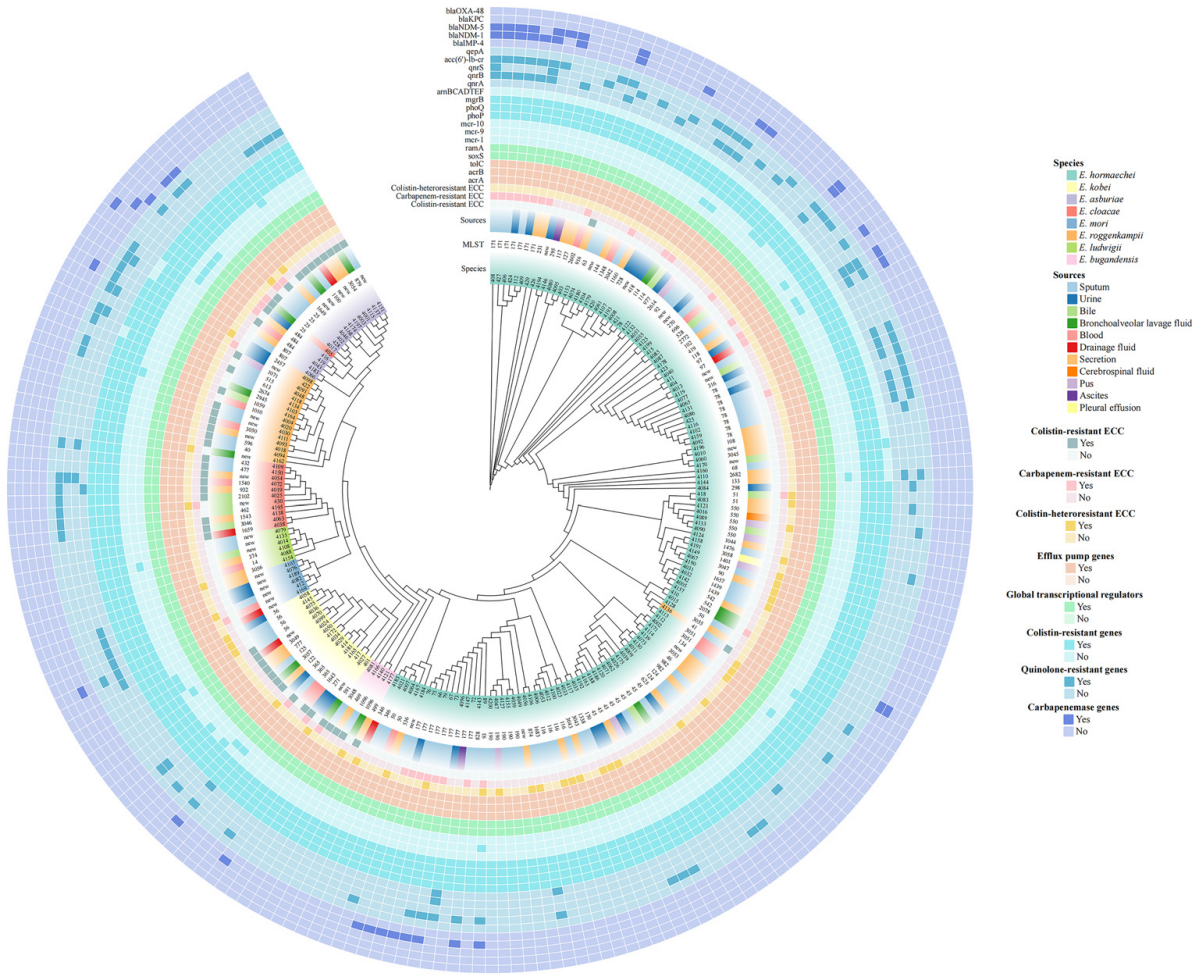
Phylogenetic tree of 212 *Enterobacter cloacae* complex (ECC) isolates based on core genome single nucleotide polymorphisms (cg-SNPs). The clades, species, multilocus sequence typing (MLST), strain sources, antimicrobial resistance profiles (colistin resistance, colistin heteroresistance and carbapenem resistance) and resistance-associated genes for major antibiotics (colistin, quinolones and carbapenems) were depicted in concentric circles.

**Figure 2:**
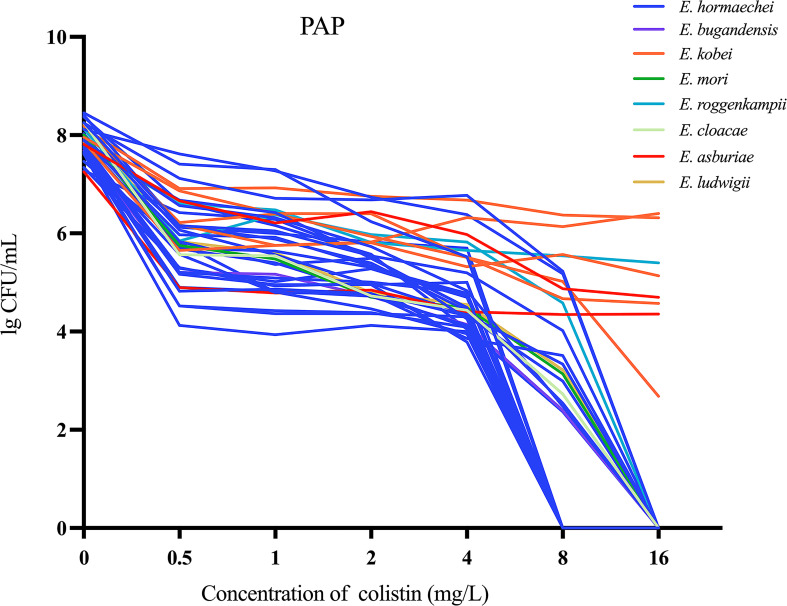
Population analysis profiles (PAP) were performed on 40 colistin-heteroresistant isolates.

Of the 212 ECC strains that were analyzed, 17.9% (38/212) were classified as carbapenem-resistant ECC (CRECC). Additionally, 22.6% (48/212) exhibited fully resistant to colistin, while the remaining strains were either sensitive or demonstrated the skip well phenomenon (**Figure 1**). The PAP confirmation analysis identified 24.4% (40/164) of the remaining strains as colistin-heteroresistant isolates, which accounted for 18.9% (40/212) of all strains (**Figure 2**). In terms of the sources of all strains, sputum constituted the highest proportion at 30.7% (65/212), followed by secretions at 18.9% (40/212), urine at 9.9% (21/212), blood at 6.6% (14/212), bile at 6.1% (13/212) and bronchoalveolar lavage fluid 5.7% (12/212). The remaining sources were made up of smaller proportions. Notably, 14.6% (31/212) of the strains were isolated from specimens collected from the sterile sites of these patients (**Figure 1**). ECC4158 transitioned from low resistance (MIC = 4 mg/L) to sensitivity (MIC = 2 mg/L). Furthermore, Significant decreases in the MIC of colistin for ECC4046 (from MIC > 128 mg/L to MIC = 4 mg/L) and ECC4075 (from MIC > 128 mg/L to MIC = 1 mg/L) were observed in passage studies of 26 resistant subpopulations. The remaining resistant subpopulations maintained stable heteroresistance after passaging (**Supplementary Figure S1**).

**Figure 3:**
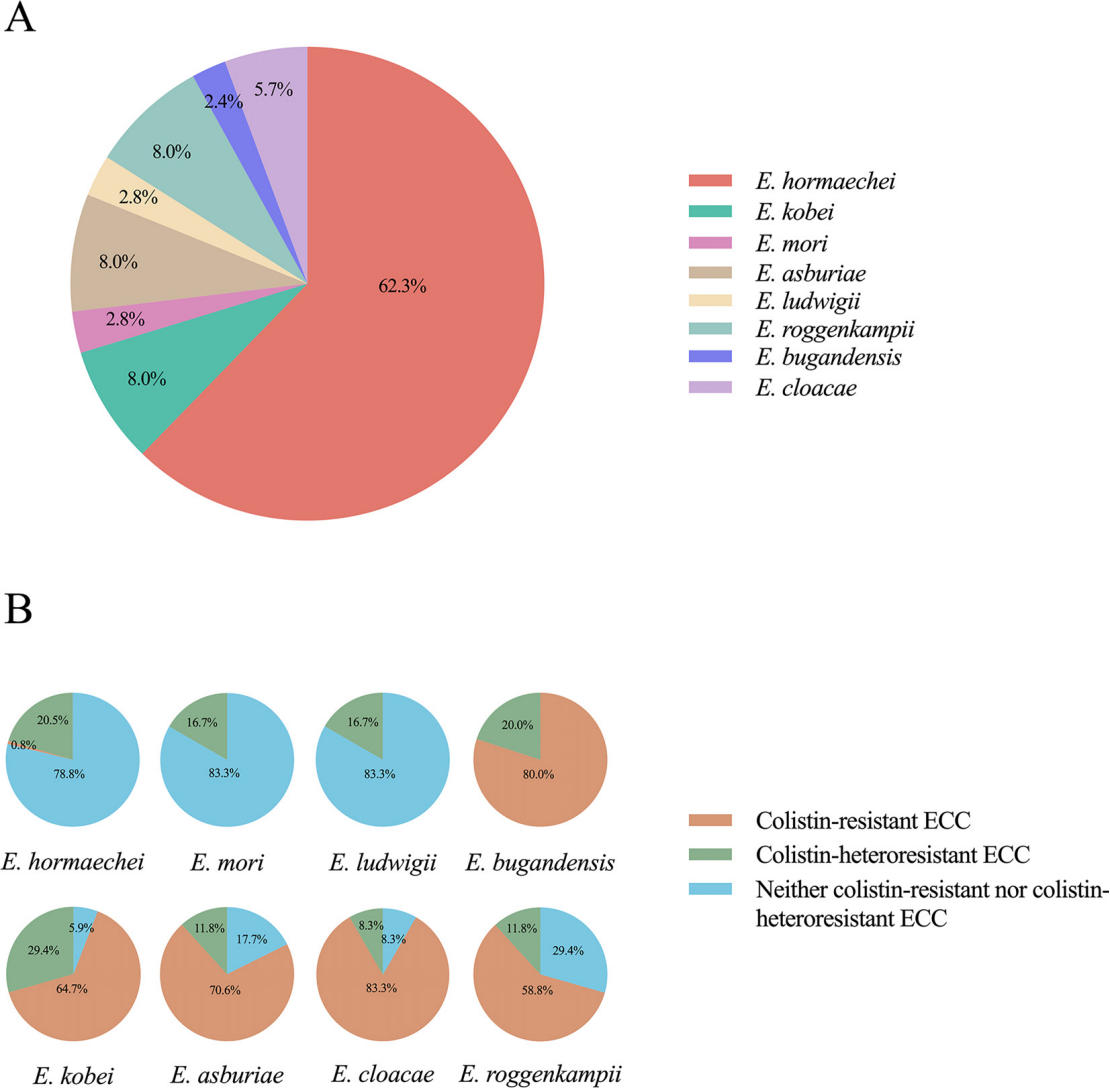
Species classification within the *Enterobacter cloacae* complex (ECC) and antimicrobial resistance and heteroresistance analysis. **(A)** Different colors represented different ECC species and their proportion. **(B)** The proportion of colistin-resistant and colistin-heteroresistant isolates within each species.

A total of eight different ECC species were identified in our study: *Enterobacter hormaechei* (62.3%; 132/212), *Enterobacter kobei* (8.0%; 17/212), *Enterobacter asburiae* (8.0%; 17/212), *Enterobacter roggenkampii* (8.0%; 17/212), *Enterobacter cloacae* (5.7%; 12/212), *Enterobacter mori* (2.8%; 6/212), *Enterobacter ludwigii* (2.8%; 6/212) and *Enterobacter bugandensis* (2.4%; 5/212) (**Figures 1**, **3A**). Of the most prevalent species*, Enterobacter hormaechei*, only one strain (0.8%; 1/132) was fully resistant to colistin. Conversely, a significant proportion of colistin-resistant ECC isolates were observed in the less prevalent species: *Enterobacter bugandensis* (80.0%; 4/5), *Enterobacter kobei* (64.7%; 11/17), *Enterobacter asburiae* (70.6%; 12/17), *Enterobacter cloacae* (83.3%; 10/12) and *Enterobacter roggenkampii* (58.8%; 10/17). No colistin-resistant ECC isolates were found among *Enterobacter mori* and *Enterobacter ludwigii* (**Figure 3B**).

### 3.2 General characteristics of colistin-heteroresistant isolates

The highest proportions of colistin-heteroresistant isolates among the various ECC species were observed in *Enterobacter kobei* (29.4%), followed by *Enterobacter hormaechei* (20.5%) and *Enterobacter bugandensis* (20.0%) (**Figure 3B**). Interestingly, the colistin heteroresistance level varied among the different ECC species: *Enterobacter kobei*, *Enterobacter asburiae*, *Enterobacter roggenkampii* and *Enterobacter bugandensis* showed high levels of heteroresistance within their resistant subpopulations (MIC ≥ 128 mg/L), except for ECC4098. In contrast, *Enterobacter hormaechei*, *Enterobacter cloacae* and *Enterobacter ludwigii* exhibited low levels of heteroresistance in their resistant subpopulations (MIC ≤ 16 mg/L), with only the ECC4082 (*Enterobacter mori*) showing a MIC of 32 mg/L (**Supplementary Table S2**).

### 3.3 Phylogenetics and comparative genomic analysis

**Figure 4:**
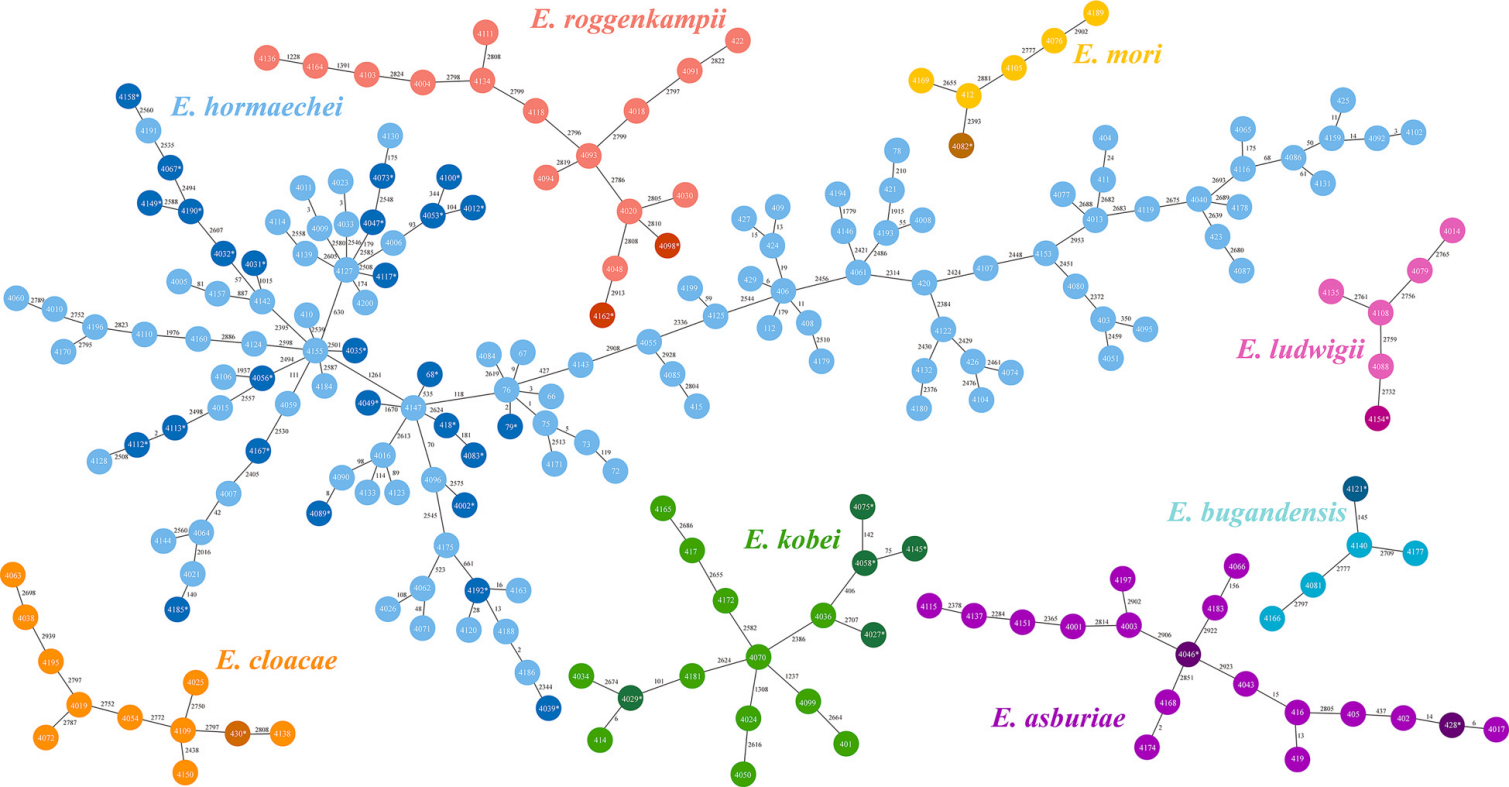
Minimum spanning tree based on core genome multilocus sequence typing (cg-MLST). The Allele differences between isolates were indicated numerically. Each color in those trees represented different species within the *Enterobacter cloacae* complex (ECC). Dark colors denoted colistin-heteroresistant isolates within each species, while light colors denoted non-colistin heteroresistant isolates.

29 distinct sequence types (STs) were identified among the 40 colistin-heteroresistant isolates, with 5 isolates belonging to novel STs. Notably, 75% (3/4) of all ST116 and ST56 strains within 212 ECC strains were heteroresistant to colistin (**Figure 1**, **Supplementary Table S2**). Furthermore, our study revealed 113 different STs among the 212 ECC strains, with 35 strains belonging to novel STs. ST177 (4.2%; 9/212), ST45 (4.2%; 9/212), ST78 (3.8%; 8/212) and ST171 (3.3%; 7/212) were the most common STs (**Figure 1**). According to the cg-MLST results, strains of ST177 showed differences in 1 to 119 alleles. Except for ST45 strains, ST78 and ST171 strains displayed results similar to those of ST177 strains (**Figure 4**). Furthermore, phylogenetic analysis based on cg-SNPs and ANI analysis revealed similarities among these strains (**Figure 1**; **Supplementary Figure S2**). These results suggest potential clonal dissemination within the ST177, ST78 and ST171 strains, all of which were categorized under *Enterobacter hormaechei* in this region. Notably, The ST177 strains were particularly likely to undergo clonal dissemination between neonatal wards (**Supplementary Table S3**).

According to WGS and previous Sanger sequencing data from our team, all 212 strains carried colistin-resistant genes (*mgrB*, *phoP*, *phoQ*), transcriptional regulators (*ramA, soxS*) associated with colistin resistance or heteroresistance and efflux pump genes (*acrA*, *acrB*, *tolC*). However, the *arnBCADTEF* operon was present in only 72.2% (153/212) of all strains. Notably, all 40 colistin-heteroresistant isolates possessed the *arnBCADTEF* operon. Additionally, some strains harbored plasmid genes potentially associated with colistin resistance, including *mcr-9* (7.5%; 16/212) and *mcr-10* (4.2%; 9/212). None of the strains carried *mcr-1*. Quinolone-resistant genes were detected in 42.0% (89/212) of all strains, with *acc(6’)-lb-cr* (32.1%; 68/212) and *qnrB* (17.0%; 36/212) being the most prevalent. Furthermore, 89.5% (34/38) of the CRECC isolates were identified as carbapenemase producers. Specifically, 29 isolates carried the *bla*_NDM-1_, 9 carried the *bla*_NDM-5_, 4 carried the *bla*_IMP-4_ and 1 carried the *bla*_KPC_. Notably, ECC410 harbored both the *bla*_NDM-1_ and *bla*_IMP-4_ (**Figure 1**).

### 3.4 Risk factors and clinical outcomes of infections caused by colistin-heteroresistant isolates

Most patients with colistin-heteroresistant ECC infections were aged 60 years or older (52.5%; 21/40) and predominantly from the surgical ward (52.5%; 21/40). The isolates primarily originated from the respiratory tract (45.0%; 18/40), with the most prevalent comorbidity for patients with colistin-heteroresistant ECC infections being respiratory diseases (55.0%; 22/40). Additionally, 80% of patients with colistin-heteroresistant ECC infections had received cephalosporin antibiotics within the past three months. Patients undergoing surgical treatment (65.0%; 26/40), urinary catheterization (52.5%; 21/40) and mechanical ventilation (35.0%; 14/40) were significantly more likely to be infected with colistin-heteroresistant ECC according to observations of invasive procedures within the past month and surgery within the past 6 weeks (**Table 1**). The infection of ST116 and ST56 strains, the use of mechanical ventilation and the exposure to cephalosporin antibiotics were all highly correlated (P < 0.05) with colistin-heteroresistant ECC infections in univariate analysis (**Table 1**). The infection of ST116 and ST56 strains as well as exposure to cephalosporin antibiotics were independent risk factors for colistin-heteroresistant ECC infections by further multivariate analysis (**Table 2**). However, no significant differences in clinical outcomes were observed between the two groups (**Table 1**).

**Table 1:** Univariate analysis of clinical characteristics of patients infected with colistin-heteroresistant *Enterobacter cloacae* complex (ECC).

Variables	COL-HR	Non-COL-HR	Univariate analysis	
	n = 40	n = 172	P-value	OR (95%CI)
Demographics, n (%)				
Male	20 (50.0)	110 (64.0)	0.105	0.564 (0.282-1.128)
Age ( ≥ 60)	21 (52.5)	88 (51.2)	0.879	1.055 (0.530-2.101)
Admission to ICU	3 (7.5)	23 (13.4)	0.315	0.525 (0.150-1.844)
Admission to the surgical ward	21 (52.5)	70 (40.7)	0.177	1.611 (0.807-3.215)
Admission to the medical ward	9 (22.5)	55 (32.0)	0.243	0.618 (0.275-1.386)
MLST, n (%)				
ST116	3 (7.5)	1 (0.6)	**0.024**	13.865 (1.403-137.038)
ST177	1 (2.5)	8 (4.7)	0.550	0.526 (0.064-4.327)
ST190	1 (2.5)	4 (2.3)	0.948	1.077 (0.117-9.904)
ST25	1 (2.5)	3 (1.7)	0.753	1.444 (0.146-14.261)
ST365	1 (2.5)	2 (1.2)	0.529	2.179 (0.193-24.647)
ST45	1 (2.5)	8 (4.7)	0.550	0.526 (0.064-4.327)
ST550	1 (2.5)	4 (2.3)	0.948	1.077 (0.117-9.904)
ST56	3 (7.5)	1 (0.6)	**0.024**	13.865 (1.403-137.0381)
Sources, n (%)				
Urinary tract	6 (15.0)	21 (12.2)	0.634	1.269 (0.476-3.383)
Gastrointestinal system	5 (12.5)	15 (8.7)	0.464	1.495 (0.510-4.387)
Skin and soft tissue	9 (22.5)	47 (27.3)	0.534	0.772 (0.342-1.743)
Respiratory tract	18 (45.0)	76 (44.2)	0.926	1.033 (0.517-2.064)
Bloodstream	2 (5.0)	12 (7.0)	0.652	0.702 (0.151-3.267)
Underlying diseases and conditions, n (%)				
**Hypertension**	13 (32.5)	44 (25.6)	0.375	1.401 (0.665-2.951)
Diabetes mellitus	6 (15.0)	37 (21.5)	0.359	0.644 (0.251-1.650)
Hypoproteinemia	15 (37.5)	64 (37.2)	0.973	1.012 (0.497-2.061)
Severe anemia	1 (2.5)	4 (2.3)	0.948	1.077 (0.117-9.904)
Sepsis	1 (2.5)	5 (2.9)	0.889	0.856 (0.097-7.539)
Chemoradiotherapy	2 (5.0)	13 (7.6)	0.573	0.644 (0.139-2.973)
Solid tumor	7 (17.5)	33 (19.2)	0.806	0.893 (0.363-2.197)
Cardiovascular diseases	14 (35.0)	67 (39.0)	0.643	0.844 (0.411-1.731)
Neurological diseases	16 (40.0)	68 (39.5)	0.957	1.020 (0.505-2.058)
Kidney diseases	12 (30.0)	44 (25.6)	0.569	1.247 (0.584-2.661)
Hepatobiliary and pancreatic diseases	12 (30.0)	54 (31.4)	0.864	0.937 (0.443-1.981)
Gastrointestinal diseases	17 (42.5)	71 (41.3)	0.888	1.051 (0.524-2.110)
Respiratory diseases	22 (55.0)	100 (58.1)	0.718	0.880 (0.440-1.759)
Hematological diseases	4 (10.0)	14 (8.1)	0.704	1.254 (0.390-4.035)
Antimicrobial exposure within the past 3 months, n (%)				
Cephalosporins	32 (80.0)	107 (62.2)	**0.037**	2.430 (1.056-5.594)
Carbapenems	7 (17.5)	33 (19.2)	0.806	0.893 (0.363-2.197)
Fluoroquinolones	8 (20.0)	41 (23.8)	0.605	0.799 (0.341-1.870)
Glycopeptides	2 (5.0)	8 (4.7)	0.925	1.079 (0.220-5.286)
Tetracyclines	2 (5.0)	9 (5.2)	0.952	0.953 (0.198-4.592)
Penicillins	20 (50.0)	100 (58.1)	0.351	0.720 (0.361-1.435)
Invasive procedures within the past month, n (%)				
Surgery within the past 6 weeks	26 (65.0)	90 (52.3)	0.150	1.692 (0.827-3.460)
Mechanical ventilation	14 (35.0)	34 (19.8)	**0.041**	2.186 (1.032-4.628)
Drainage tube insertion	13 (32.5)	44 (25.6)	0.375	1.401 (0.665-2.951)
Urinary catheterization	21 (52.5)	83 (48.3)	0.629	1.185 (0.595-2.360)
Tracheal cannula	8 (20.0)	28 (16.3)	0.573	1.286 (0.536-3.082)
Arterial and venous catheterization	6 (15.0)	38 (22.1)	0.322	0.622 (0.243-1.593)
Gastrointestinal catheterization	13 (32.5)	56 (32.6)	0.994	0.997 (0.478-2.079)
Bronchoscopy	8 (20.0)	28 (16.3)	0.573	1.286 (0.536-3.082)
Clinical outcomes				
Hospital stay, median (IQR)	14.50(7.00-29.00)	19.50(11.00-33.75)	0.662	0.997 (0.984-1.010)
Disease progression, n (%)	6 (15.0)	31 (18.0)	0.651	0.803 (0.310-2.078)

Data are expressed as the number of patients (percentage) for categorical variables and the mean ± standard deviation or median (IQR) for continuous variables as appropriate.

COL-HR, colistin-heteroresistant *Enterobacter cloacae* complex; Non-COL-HR, Non-colistin heteroresistant *Enterobacter cloacae* complex; OR, odds ratio; CI, confidence interval; ICU, intensive care unit; MLST, multilocus sequence typing; ST, sequence type; IQR, interquartile range.

Bold face indicated significant values (P < 0.05).

**Table 2:** Multivariate analysis of clinical characteristics of patients infected with colistin-heteroresistant *Enterobacter cloacae* complex (ECC).

Variables	COL-HR	Non-COL-HR	Multivariate Analysis	
	n = 40	n = 172	P-value	OR (95%CI)
Cephalosporins	32 (80.0)	107 (62.2)	**0.024**	2.967 (1.157-7.605)
Mechanical ventilation	14 (35.0)	34 (19.8)	0.069	2.090 (0.945-4.622)
ST116	3 (7.5)	1 (0.6)	**0.012**	20.885 (1.947-224.069)
ST56	3 (7.5)	1 (0.6)	**0.012**	23.140 (2.008-266.707)

COL-HR, colistin-heteroresistant *Enterobacter cloacae* complex; Non-COL-HR, Non-colistin heteroresistant *Enterobacter cloacae* complex; OR, odds ratio; CI, confidence interval; ST, sequence type.

Bold face indicated significant values (P < 0.05).

### 3.5 Molecular mechanisms of colistin-heteroresistant isolates

Non-synonymous substitutions in *phoQ* among colistin-heteroresistant isolates: R423C (n = 5), L9 frameshift (n = 1), N90D, N150D (n = 1) and N90D (n = 1). Additionally, a unique C16S variation in *mgrB* was identified in ECC4162. No mutation in *phoP* was detected across all isolates (**Supplementary Table S2**). Efflux pump inhibition assays demonstrated that the MICs of these resistant subpopulations decreased by 8-fold or greater in the presence of CCCP. Specifically, 9 resistant subpopulations exhibited MIC reductions of 128-fold or greater, while the remaining showed reductions ranging from 8 to just below 128-fold. Furthermore, MICs of 7 parental strains decreased by 4-fold or more. Nevertheless, only 44.0% (12/26) of these resistant subpopulations had MICs that decreased by 8-fold or more after exposure to PAβN. These findings indicated that the efflux pump activity was markedly inhibited by EPIs, especially for CCCP (**Supplementary Table S4**).

**Figure 5:**
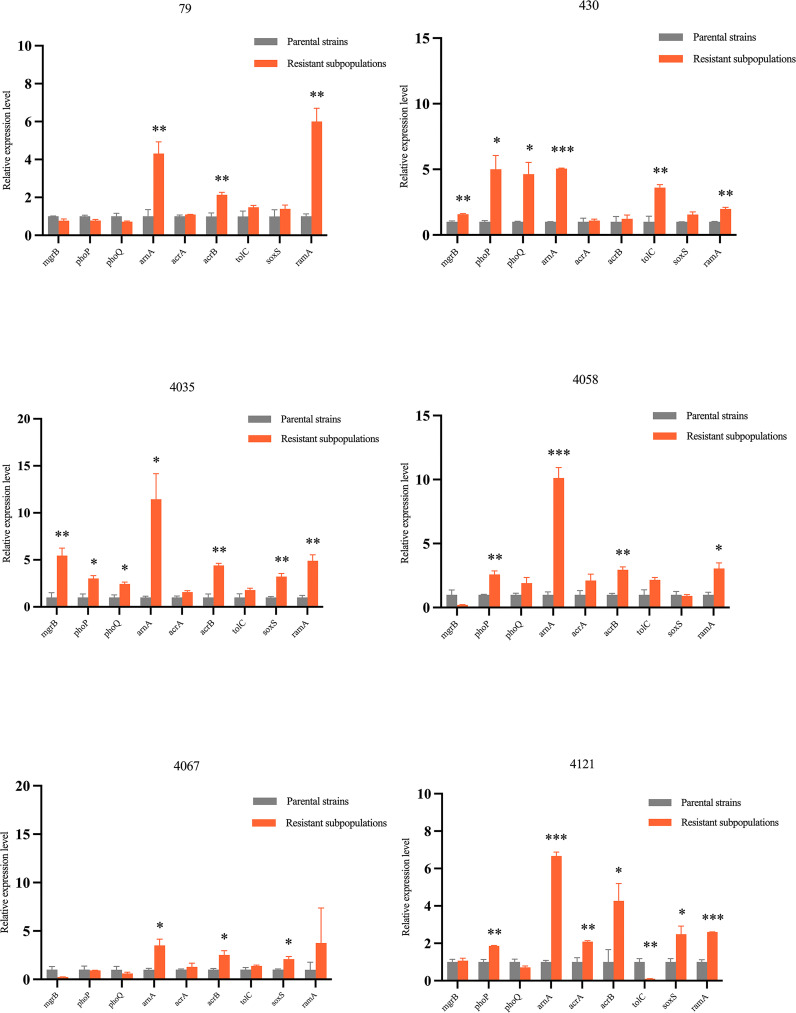
Gene expression levels of colistin-heteroresistant parental strains and resistant subpopulations. The *rpoB* gene was used as an internal control to normalize the data. Results were expressed as the mean ± SEM of three independent experiments. Statistical significance was assessed using Student's t-test, with p-values < 0.05 considered statistically significant; *p < 0.05, **p < 0.01, ***p < 0.001. Data were analyzed using GraphPad Prism 9.0 statistical software.

**Figure 6:**
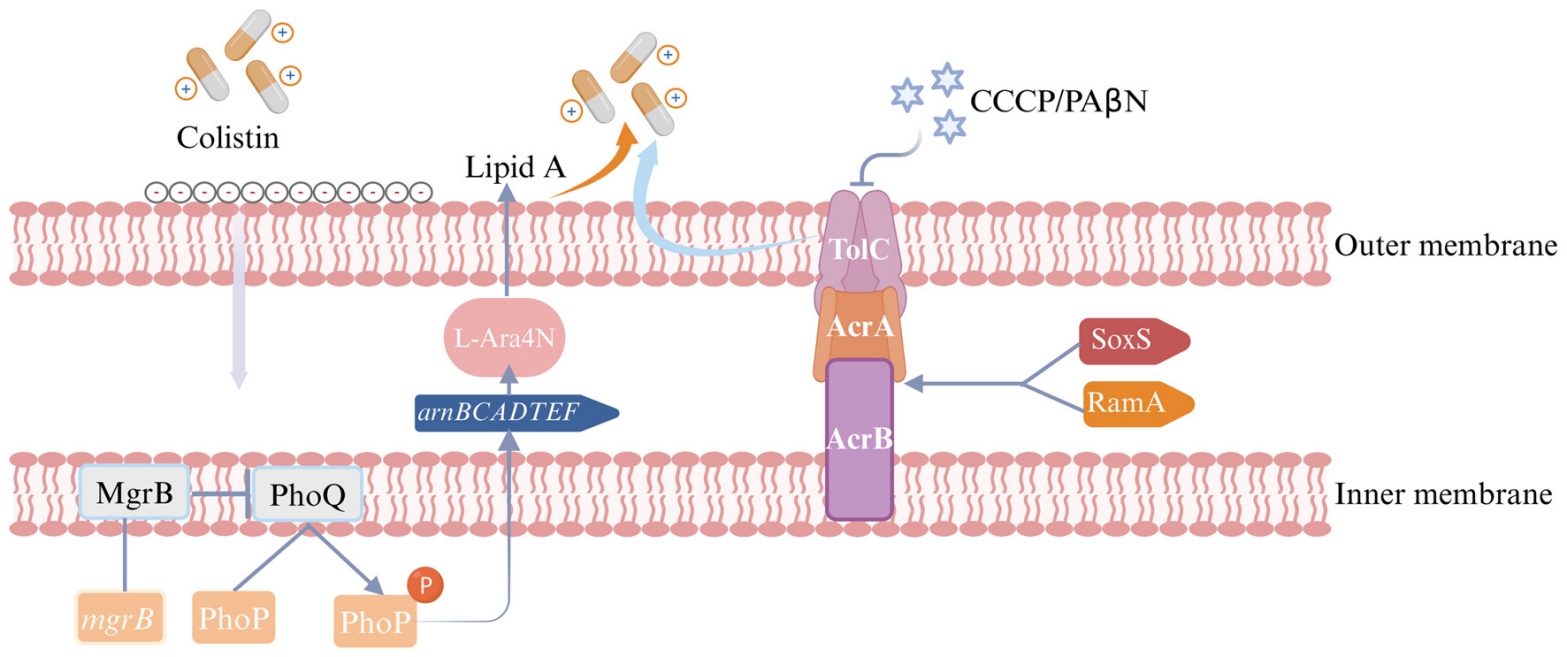
Colistin heteroresistance mechanism in *Enterobacter cloacae* complex (ECC). The figure was created with Biorender.

RT-qPCR experiments revealed that *arnA* was significantly overexpressed in resistant subpopulations as compared to their parental strains, with statistical significance observed across all 6 strains. High levels of *ramA* and *acrB* expression were detected in 5 resistant subpopulations and elevated *phoP* expression was observed in 4 resistant subpopulations. Furthermore, *soxS* overexpression was found in 50% of resistant subpopulations. Conversely, overexpression of *phoQ*, *acrA* and *tolC* with statistical significance was observed in only a small number of resistant subpopulations. Notably, no resistant subpopulations exhibited a statistically significant decrease in *mgrB* expression levels (**Figures 5**, **6**).

## 4 Discussion

ECC strains have posed a significant threat to public health as one of the “ESKAPE” pathogens (Liu et al., 2021a). The difficulty of identifying heteroresistance in conventional susceptibility testing methods exacerbates the neglect of colistin heteroresistance in ECC. Therefore, addressing the growing threat of heteroresistance is an urgent necessity. The study demonstrated that colistin heteroresistance in ECC occurred at a rate of 24.4% in non-resistant isolates, with significant species-dependent differences in the prevalence rates and levels of colistin heteroresistance. Infections with the ST116 and ST56 strains were strongly associated with colistin heteroresistance in ECC. Clonal dissemination of ST177, ST78 and ST171 strains may exist in this region and ST177 strains were particularly likely to undergo clonal dissemination between neonatal wards. Multiple genes promoted colistin heteroresistance through a complex interplay. It is important to emphasize that standardized definitions and methods are crucial for improving research reproducibility and clinical diagnostics.

The study showed that 22.6% of ECC strains were fully resistant to colistin. A higher prevalence rate of 34.5% for full colistin resistance among 190 *Enterobacter* spp. isolates in 2020 was reported in China and this proportion has been increasing over the years (Liao et al., 2022). These findings highlighted the need to monitor the prevalence of colistin resistance in ECC and the urgent need for alternative treatment strategies. In this region, the colistin heteroresistance prevalence rate accounting for all strains was 18.9%, consistent with the findings of Guérin et al., who reported the colistin heteroresistance prevalence rate of 18.5% (Guerin et al., 2016). However, this rate was lower than the 27.5% observed in a study from Japan (Fukuzawa et al., 2023). A possible explanation for this observation is that colistin was not routinely used in this region and most patients weren’t exposed to colistin before infection, which posed a potential threat. Additionally, our initial screening using antibiotic discs and electronic test strips may be the reason for the relatively low prevalence. Although these methods were simpler than PAP analysis and more suitable for large-scale screening, they may be less effective in detecting low-frequency resistant subpopulations due to the plate’s lower cell density. Even though PAP analysis is widely regarded as the gold standard for characterizing heteroresistant isolates, it is costly, time-consuming and cannot efficiently test samples. Therefore, developing more direct, rapid and clinically applicable methods for identifying heteroresistance represents a promising direction for future research. The MICs of ECC4046 and ECC4075 significantly decreased after 50 generations. This instability may arise from the acquisition of genetically stable but costly resistance mutations, with the absence of antibiotics driving the selection of compensatory mutations that reduce the cost and loss associated with resistance; Or the emergence of resistant subpopulations may be attributed to unstable and costly gene tandem amplifications and in the absence of antibiotic selection pressure, these amplifications could be lost, leading to the restoration of bacterial susceptibility (Hjort et al., 2016; Nicoloff et al., 2019). Additionally, we speculate that colistin-resistant subpopulations could replicate under continuous antibiotic pressure. These subpopulations would become dominant and evolve into fully resistant isolates, leading to antibiotic treatment failure (Band et al., 2016; Li et al., 2022).

*Enterobacter hormaechei* was the predominant species identified in our research. Among the high proportion (62.3%) of *Enterobacter hormaechei*, only one strain exhibited full colistin resistance. In contrast, a high proportion of colistin-resistant ECC isolates were identified among other species, excluding *Enterobacter mori* and *Enterobacter ludwigii*. To our knowledge, it was the first observation of such a phenomenon. Interestingly, a considerable proportion of colistin heteroresistance was observed in *Enterobacter hormaechei* in which almost all strains were susceptible to colistin and the proportion of the different species varied significantly. According to another study, cluster dependence was present for colistin heteroresistance in ECC (Guerin et al., 2016). The resistant subpopulations demonstrated different colistin heteroresistance levels among ECC species. Doijad et al. pointed out that allele variations in the *phoPQ* or *mgrB* loci across different ECC species could lead to differences in colistin resistance and heteroresistance levels (Doijad et al., 2023). Consequently, it is imperative to enhance colistin resistance and heteroresistance monitoring across different ECC species.

A noteworthy discovery from our study was that a high proportion (75%) of colistin heteroresistant isolates was observed among the ST116 and ST56 strains, with ST116 strains being the major lineages of carbapenem-resistant *Enterobacter* spp. in other region of China (Zhu et al., 2023). According to clonal diversity analysis of all ECC strains, the most common STs were ST177, ST45, ST78 and ST171. Strains of ST177, ST78 and ST171 may be linked to clonal dissemination in this region and we should remain vigilant about the spread of ST177 strains in neonatal wards. Notably, all these STs fell within *Enterobacter hormaechei*. This phenomenon suggested that different ECC species might possess distinct evolutionary or transmission mechanisms. Furthermore, our study identified ST177 and ST171 as the predominant STs among CRECC isolates. Other studies reported that ST93, ST171 and ST145 were the main STs among CRECC isolates (Chen et al., 2021); ST171 being the most common clone among carbapenem-resistant *Enterobacter* spp. dominated by *E. xiangfangensis* (Chavda et al., 2016). The observed differences may stem from geographical and genetic diversity, as well as the limited number of CRECC isolates in this study. A high prevalence of *bla*_NDM-1_ was identified in 76.3% of the 38 CRECC isolates, aligning with other study reporting a 74% carriage rate of *bla*_NDM-1_ in CRECC isolates (Jia et al., 2018). Additionally, *bla*_NDM-5_ was exclusively found in *Enterobacter hormaechei*.

The study found that elderly patients were more likely to be infected with colistin-heteroresistant ECC isolates. This phenomenon could explained by a higher prevalence of underlying health conditions and diminished physiological function in elderly patients (Jia et al., 2020). Patients infected with colistin-heteroresistant ECC isolates were mainly from surgical wards, which may be related to the presence of wounds and frequent invasive procedures common in these wards. Colistin-heteroresistant ECC primarily originated from the respiratory tract and the most prevalent comorbidity among patients was respiratory diseases. The frequent exposure of the respiratory tract to antibiotic selective pressure and its complex host microenvironment may promote the survival and proliferation of resistant subpopulations. Mechanical ventilation exacerbated this phenomenon. Infections with the strains of ST116 and ST56 were independent risk factors for colistin-heteroresistant ECC infections, so it is essential to warrant vigilance regarding their prevalence. Furthermore, prolonged use of cephalosporin antibiotics could disrupt the intestinal environment, resulting in genetic changes that promote the development of colistin heteroresistance.

This study found that high-level expression of the *arnA* gene, which is part of the *arnBCADTEF* operon, was observed in 100% (6/6) of the resistant subpopulations as compared to their parental strains. This was inconsistent with previous studies, which indicated that the expression levels of *arnA* genes in colistin-heteroresistant isolates remained unchanged (Telke et al., 2017). Additionally, elevated expression of *phoP* and *phoQ* was detected in resistant subpopulations of ECC430 and ECC4035. These findings indicated that the mechanisms of colistin heteroresistance in ECC were attributed to the PhoPQ TCS, which upregulated the expression of *arnBCADTEF* operon to synthesize L-Ara4N and incorporate it into lipid A, a component of lipopolysaccharide (LPS). This reduced the negative charge on LPS and decreased colistin binding to the bacterial outer membrane. Consequently, the isolates showed heteroresistance to colistin (Guerin et al., 2016; Huang et al., 2019). For resistant subpopulations of ECC4058 and ECC4121, the expression level of *phoP* was significantly elevated, while concurrent high expression of *phoQ* was not observed. The reason for the increased expression of *phoP* which upregulates the *arnA*, warrants further investigation. Conversely, elevated expression levels of *phoP* and *phoQ* were not observed in resistant subpopulations of ECC79 and ECC4067. Both ECC79 and ECC4067 harbored the “R423C” mutation in *phoQ*, which may be linked to colistin heteroresistance. According to our study, *mgrB* which could negatively control the PhoPQ TCS may play a minimal role in determining colistin heteroresistance in ECC. The presence of CCCP significantly reduced the MICs of all resistant subpopulations. We speculated that the heteroresistance to colistin in ECC may be related to the activity of the efflux pump. Our research found that *acrB* was highly expressed in 83.3% of the resistant subpopulations, while elevated expression levels of *acrA* or *tolC* were observed in only one resistant subpopulation. Although the products of *acrA*, *acrB*, and *tolC* genes worked together to form the AcrAB-TolC pump, these genes were not genetically clustered (Li et al., 2015). Huang et al. previously demonstrated that knockout of *acrB* was insufficient to eliminate colistin heteroresistance, whereas *tolC* was essential for this phenotype (Huang et al., 2019). Thus, we hypothesized that *acrB* may promote colistin heteroresistance without being strictly necessary, whereas the *tolC* might promote colistin heteroresistance through other mechanisms. Another study observed that the inactivation of the *acrA* in *Enterobacter cloacae* resulted in increased susceptibility to various antibiotics (Perez et al., 2012). Consequently, the role of *acrA* in colistin heteroresistance warrants further investigation. Overall, the impact of different efflux pump genes on antibiotic heteroresistance varied and promiscuity or redundancy for these genes with other efflux pump genes could impact colistin heteroresistance. 83.3% of the resistant subpopulations exhibited high levels of *ramA* expression, while half of the resistant subpopulations showed elevated *soxS* expression in this study. These genes could enhance the bacterial ability to expel antibiotics by upregulating the expression of the AcrAB-TolC efflux pump genes, contributing to the development of colistin heteroresistance (Blair et al., 2015). In conclusion, colistin heteroresistance mechanisms are complex and further research to elucidate the mechanisms is essential.

Although infections with colistin-heteroresistant isolates in this study did not significantly increase hospitalization time or disease progression, more studies suggest that strain heteroresistance can lead to treatment failure and poor prognosis. For example, a study found colistin heteroresistance was associated with treatment failure (Band et al., 2016). The frequency and resistance levels of heteroresistance differ across studies, which may explain the variation in results (Andersson et al., 2019). At present, there is limited evaluation of antimicrobial treatments for colistin-heteroresistant CRECC isolates. Colistin combination therapy may be a promising strategy for overcoming colistin heteroresistance. For example, the combination of colistin with tetracyclines (tigecycline or minocycline) or aminoglycosides (amikacin or gentamicin) has shown strong bactericidal effects against colistin-heteroresistant carbapenem-resistant *Klebsiella pneumoniae* (Wang et al., 2022). Notably, three recently approved β-lactam/β-lactamase inhibitor combinations−CAZ/AVI, meropenem/vaborbactam (MEV) and imipenem-cilastatin/relebactam (ICR), which fail to inhibit MBLs, limiting their clinical use (Zhang et al., 2023). Cefepime–taniborbactam which is in clinical trials, significantly improved the antibacterial effect of cefepime against multidrug-resistant Gram-negative bacteria (Karlowsky et al., 2024). It may be useful in the future. Overall, the treatment of colistin-heteroresistant ECC infection needs to be further validated in animal models and clinical studies.

In conclusion, this study underscored the high prevalence of colistin heteroresistance in ECC, revealing notable species-dependent variations in both the prevalence rates and levels of heteroresistance. These variations illustrated the necessity for tailored antimicrobial stewardship programs. For high-risk patients infected with colistin-heteroresistant CRECC isolates, such as those with prior cephalosporin exposure, combination therapy involving colistin and other agents should be prioritized to target potential resistant subpopulations. Notably, the strains of ST116 and ST56 exhibited a high correlation with colistin heteroresistance. Multiple genes promoted colistin heteroresistance through a complex interplay. Consequently, further investigation into the mechanisms of colistin heteroresistance, as well as continuous monitoring and molecular characterization of these pathogens are required. Several limitations of this study should be acknowledged. As a single-center retrospective study, the epidemiology and resistance mechanisms of the isolates may not be representative of those in other regions. Furthermore, current detection methods, such as PAP analysis, are inherently limited. Consequently, developing more direct, rapid and clinically applicable methods for detecting heteroresistance is crucial to optimizing antimicrobial management and enhancing patient outcomes.

## Data Availability

The datasets presented in this study can be found in online repositories. The names of the repository/repositories and accession number(s) can be found in the article/**Supplementary Material**.
