# Supplementary material for: Clinical characteristics, molecular epidemiology and mechanisms of colistin heteroresistance in Enterobacter cloacae complex
Source: Front Cell Infect Microbiol. 2025 Mar 6;15:1536058. doi: 10.3389/fcimb.2025.1536058 (PMC11922889; doi:10.3389/fcimb.2025.1536058)
Supplement: Supplementary file 1 [file Table1.docx]

**Supplementary Table S1** Primer sequences used in this work.

| Primers | Sequence (5' → 3') |
| --- | --- |
| Used for *Enterobacter hormaechei*; PCR | |
| *mgrB*-F | GGTCTTAAGACGGAGTGTGGA |
| *mgrB*-R | CACTCAGCGTAAAAATGGCCT |
| *phoP*-F | TTATAAAGGGAGAAGTGATG |
| *phoP*-R | TGACGCAAAATCCCTTTCAT |
| *phoQ1*-F | ATGACGTGATCACCACCGTG |
| *phoQ1*-R | TCTGCTGTGAAATACGGCTG |
| *phoQ2*-F | AACATGCCGCAGTTAACCAT |
| *phoQ2*-R | CTCCCTGGCTGCGAGATCCG |
| Used for *Enterobacter kobei* and *Enterobacter bugandensis*; PCR | |
| *mgrB*-F | CACCTTAAGAAAAAATGCGTGCTAC |
| *mgrB*-R | GGTCTTTAGACGGAGTGTGGA |
| *phoP*-F | CATATTCTGGGAGAAAAGATGATGC |
| *phoP*-R | ATGGCGCAAAATCTGTCTCATTTAG |
| *phoQ1*-F | TCGCATTTTTCACATAACGGGTTAA |
| *phoQ1*-R | GCGAGCTTGAGGAACACCATC |
| *phoQ2*-F | CTTTTCAGCAGGCGATTGAGAT |
| *phoQ2*-R | TACCTGTTTGAATTACGCTAAATGAG |
| Used for *Enterobacter asburiae*; PCR | |
| *mgrB*-F | CACCTTGAGAAAAAATGCGTATTAC |
| *mgrB*-R | GGTTTTTAGACGGAGTGTGGAG |
| *phoP*-F | ATTATTTAGGGAGAAGAGATGATGC |
| *phoP*-R | GTGGCGTAAAATCCCTCTCATT |
| *phoQ1*-F | TCGCATTTTTCACATAACGGGTTAA |
| *phoQ1*-R | GTGAGCTGGAAGAACATCATCG |
| *phoQ2*-F | CTTTTCAGCAGGCGGTTGAGG |
| *phoQ2*-R | TACCTGTTCGAATTACGCTAAATGAG |
| Used for *Enterobacter cloacae*; PCR | |
| *mgrB*-F | ACAGGTGTTAAGACGGAG |
| *mgrB*-R | AAGGAAATCACCTTAAGAA |
| *phoP*-F | ATTCATACTATTAATTCTGGGAGAAAG |
| *phoP*-R | GGGCATAATGTGCCGCATTAG |
| *phoQ1*-F | GTGCGCGGTCAGGGCTAC |
| *phoQ1*-R | GTATTTATCGTAGCGCTCGCG |
| *phoQ2*-F | AATCGCTGGCGAAAGAGGTTC |
| *phoQ2*-R | TGCGAGATCCGCGCATTTTTC |
| Used for *Enterobacter roggenkampii*; PCR | |
| *mgrB*-F | GTTACATCAGGTCCTTAGACGG |
| *mgrB*-R | GAAGGAAATCACGTTGAGAAAAAATG |
| *phoP*-F | CTATTTATTCTGGGAGAAAAG |
| *phoP*-R | CAGAAGATGACGCAAAATC |
| *phoQ1*-F | TCAGGGCTACCTGTTTGA |
| *phoQ1*-R | GGTATTTATCATAGCGTTCG |
| *phoQ2*-F | TGGCGAAAGAGGTTCGCGAG |
| *phoQ2*-R | GCGAGATCCTCGCATTTTTCAC |
| Used for *Enterobacter mori*; PCR | |
| *mgrB*-F | CGGGTCATAAAACGGAGAGTG |
| *mgrB*-R | GAAGGAAATGACCTTAGGAAAAAATG |
| *phoP*-F | TTCATACCAATTATTTAGGGAGAAGAG |
| *phoP*-R | GGGGCAGAAAGTGTCGTGAAA |
| *phoQ1*-F | TGGTCAGGGTTACCTGTTCGA |
| *phoQ1*-R | GGTGCGGTATTTATCATAGCGT |
| *phoQ2*-F | TGGCCAAAGAGGTTCGCGAG |
| *phoQ2*-R | CGAGATCCGCGCATTTTTCAC |
| Used for *Enterobacter ludwigii*; PCR | |
| *mgrB*-F | GTTATATCGGGCCTTTAGACGG |
| *mgrB*-R | GAAGGAAATCACCTTGAGAAAAAATG |
| *phoP*-F | TTCATACCATTCATATAGGGAGAAAA |
| *phoP*-R | GGGCAGAATATGACGCAAGATC |
| *phoQ1*-F | GGTCAGGGTTATCTGTTCGAAA |
| *phoQ1*-R | GGTGCGGTATTTATCATAGCGC |
| *phoQ2*-F | TGGCGAGAGAGGTGCGCG |
| *phoQ2*-R | GCGAGATCCTCGCATTTTTCAC |
| Used for *Enterobacter hormaechei*; RT-qPCR | |
| *mgrB*-F | TACGCTGGGTAATTCTGATTATAGTG |
| *mgrB*-R | ATTGAACAAATGCCGCTAAAGAACTG |
| *phoP*-F | CGAAAGCCACACCATTGACG |
| *phoP*-R | GAACAGGTAGCCCTGACCAC |
| *phoQ-*F | TGGTCTGGAACTGGTTCGTGT |
| *phoQ-*R | CACCTCTTTGGCAAGGGATT |
| *arnA*-F | CGACATTACCGCCATCTTCACC |
| *arnA*-R | AATACCGTGTTCAGCCGCAATAC |
| *acrA*-F | TCCGAACCTCTCCAGATGACAAC |
| *acrA*-R | GCCTTGACATCACCGCCTTC |
| *acrB*-F | ACGCACCACTACACCGACAG |
| *acrB*-R | GGCAGACGAACGAACAGGAATG |
| *tolC*-F | ACCGCACGTAACAACCTCG |
| *tolC*-R | TTTGTCCGTTTTGAAACTGTCC |
| *soxS*-F | ATCGACCAGCCGTTGAACAT |
| *soxS*-R | CTGCGCTGGCGAATGTAATC |
| *ramA*-F | GTACGATATCTGCCTGCGCT |
| *ramA*-R | GCCCGACCGTGGTTTTCTT |
| Used for *Enterobacter kobei*; RT-qPCR | |
| *mgrB*-F | ACGCTGGGTGATTCTGATTGT |
| *mgrB*-R | TCCTGATCGCACATCACATTG |
| *phoP*-F | TTATCTCCATTCCGCCTTTCC |
| *phoP*-R | TGCTCACAACCTTGCCACTG |
| *phoQ-*F | GGGTTACAGCGTCAGCTTCG |
| *phoQ-*R | GATTCAGGTTCTCGGGCATTT |
| *arnA*-F | ACGGCACCTTCAGCGAGAC |
| *arnA*-R | TGGCGAGCAGGTTGGTTCC |
| *acrA*-F | GCACCAATCCAACCGCACTTC |
| *acrA*-R | CACCAGCCATTTATCGCCAATCG |
| *acrB*-F | TGGCAGACGAACGAACAGATAGG |
| *acrB*-R | TGACAAGAGCACGCACCACTAC |
| *tolC*-F | CTGACGGTAAATCGCCTGTTTCTG |
| *tolC*-R | AGCAGACGCTGATCCTGAACAC |
| *soxS*-F | CAGAGAATGTTCCGCACGGTTATG |
| *soxS*-R | AAAGGTTTGTTGCGACACATAGCC |
| *ramA*-F | TCTGCCTGCGTTACGGGTTTG |
| *ramA*-R | ACTGTGGTTCTCCTTGCGGTAC |
| Used for *Enterobacter bugandensis*; RT-qPCR | |
| *mgrB*-F | ACGCTGGGTGATTCTGATTGT |
| *mgrB*-R | TCCTGATCGCACATCACATTG |
| *phoP*-F | GGCAGGACAAAGTTGAAGTGC |
| *phoP*-R | CTGGAAAGGCGGAATGGAG |
| *phoQ-*F | GGGTTACAGCGTCAGCTTCG |
| *phoQ-*R | GATTCAGGTTCTCGGGCATTT |
| *arnA*-F | AACGGCACCTTCAGCGAGAC |
| *arnA*-R | TGGCGGGCGGGTTTATTC |
| *acrA*-F | GAATCCAACCGCACTTCTTGTTCC |
| *acrA*-R | CACCAGCCATTTATCGCCAATCG |
| *acrB*-F | TTCCTGTTCGTTCGTCTGCCAAG |
| *acrB*-R | CGTCACTTCGTCCAGCACCTTC |
| *tolC*-F | AGCCTGTTTCACGCCAATTTATAGC |
| *tolC*-R | AAGGTCGTCGTTGCCATCGG |
| *soxS*-F | ATCGACCAACCGTTGAACATTGATG |
| *soxS*-R | TGACCGTGCGGAACATTCTCTG |
| *ramA*-F | CGTCACTTCGTCCAGCACCTTC |
| *ramA*-R | GCCGCTGTAAATGCCACTTGG |
| Used for *Enterobacter cloacae*; RT-qPCR | |
| *mgrB*-F | AAATACGCTGGGTAATTCTGATTATCG |
| *mgrB*-R | TACATCCTGATCGCACATCACATTG |
| *phoP*-F | CCATCGTTGATCTCGGGTTAC |
| *phoP*-R | TTTGTCCTGCCAGCCTTCAC |
| *phoQ-*F | CGCAAAGTGGGAAAACAACC |
| *phoQ-*R | TTCACCAGCCAGGGAACATC |
| *arnA*-F | TGCCATCAGCCGTTTCCT |
| *arnA*-R | CAGCACCACGTCGCATTTT |
| *acrA*-F | GCCTGGTAATTCGGTGGTGATTTG |
| *acrA*-R | CCTCTGGCGGTCGTTCTGATG |
| *acrB*-F | CGATAAGAGCACGCACCACTACAC |
| *acrB*-R | GCTTGGCAGACGGACGAACAG |
| *tolC*-F | CTAACGGCGTAAACTCCAATGCTAC |
| *tolC*-R | GCGTCTGCTGGTCGGTCTG |
| *soxS*-F | ATCGACCAACCGTTGAACATTGATG |
| *soxS*-R | TGACCGTGCGGAACATTCTCTG |
| *ramA*-F | TCTGCCTGCGTTACGGGTTTG |
| *ramA*-R | ACTGTGGTTCTCCTTGCGGTAC |
| Used for housekeeping gene *rpoB*; RT-qPCR | |
| *rpoB*-F | AAGGCGAATCCAGCTTGTTCAGC |
| *rpoB*-R | TGACGTTGCATGTTCGCACCCATCA |

Abbreviations: PCR, polymerase chain reaction; RT-qPCR, reverse transcription quantitative PCR.

**Supplementary Table S2** The species, sources and multilocus sequence typing (MLST) of colistin-heteroresistant *Enterobacter cloacae* complex (ECC), as well as the MICs of resistant subpopulations and heteroresistance-associated gene mutations.

| Isolates | Species | Sources | MLST | MIC | *mgrB* | *phoP* | *phoQ* |
| --- | --- | --- | --- | --- | --- | --- | --- |
| 68 | *Enterobacter hormaechei* | Sputum | 93 | 8 | WT | WT | R423C |
| 79 | *Enterobacter hormaechei* | Sputum | 177 | 16 | WT | WT | R423C |
| 418 | *Enterobacter hormaechei* | Bile | 51 | 8 | WT | WT | WT |
| 4002 | *Enterobacter hormaechei* | Sputum | 134 | 4 | WT | WT | R423C |
| 4012 | *Enterobacter hormaechei* | Secretion | 116 | 8 | WT | WT | WT |
| 4031 | *Enterobacter hormaechei* | Sputum | 1657 | 4 | WT | WT | WT |
| 4032 | *Enterobacter hormaechei* | Sputum | 1439 | 8 | WT | WT | WT |
| 4035 | *Enterobacter hormaechei* | Urine | 170 | 16 | WT | WT | WT |
| 4039 | *Enterobacter hormaechei* | Secretion | 625 | 8 | WT | WT | WT |
| 4047 | *Enterobacter hormaechei* | Pus | 190 | 8 | WT | WT | WT |
| 4049 | *Enterobacter hormaechei* | Secretion | new | 8 | WT | WT | L9FS |
| 4053 | *Enterobacter hormaechei* | Sputum | 116 | 4 | WT | WT | WT |
| 4056 | *Enterobacter hormaechei* | Sputum | 874 | 8 | WT | WT | WT |
| 4067 | *Enterobacter hormaechei* | Sputum | 3047 | 8 | WT | WT | R423C |
| 4073 | *Enterobacter hormaechei* | Sputum | 982 | 4 | WT | WT | WT |
| 4083 | *Enterobacter hormaechei* | Secretion | 51 | 8 | WT | WT | WT |
| 4089 | *Enterobacter hormaechei* | Pus | 550 | 16 | WT | WT | WT |
| 4100 | *Enterobacter hormaechei* | Sputum | 116 | 4 | WT | WT | WT |
| 4112 | *Enterobacter hormaechei* | Blood | 3051 | 4 | WT | WT | WT |
| 4113 | *Enterobacter hormaechei* | Blood | 3051 | 8 | WT | WT | WT |
| 4117 | *Enterobacter hormaechei* | Sputum | 1338 | 4 | WT | WT | WT |
| 4149 | *Enterobacter hormaechei* | Pus | 1401 | 8 | WT | WT | R423C |
| 4158 | *Enterobacter hormaechei* | Sputum | 1476 | 4 | WT | WT | WT |
| 4167 | *Enterobacter hormaechei* | Secretion | 536 | 8 | WT | WT | WT |
| 4185 | *Enterobacter hormaechei* | Drainage fluid | 346 | 4 | WT | WT | WT |
| 4190 | *Enterobacter hormaechei* | Secretion | 90 | 8 | WT | WT | WT |
| 4192 | *Enterobacter hormaechei* | Urine | 45 | 8 | WT | WT | WT |
| 4027 | *Enterobacter kobei* | Urine | new | 128 | WT | WT | WT |
| 4029 | *Enterobacter kobei* | Urine | 365 | > 128 | WT | WT | WT |
| 4058 | *Enterobacter kobei* | Urine | 56 | 128 | WT | WT | WT |
| 4075 | *Enterobacter kobei* | Sputum | 56 | > 128 | WT | WT | WT |
| 4145 | *Enterobacter kobei* | Sputum | 56 | > 128 | WT | WT | WT |
| 428 | *Enterobacter asburiae* | Sputum | 25 | > 128 | WT | WT | WT |
| 4046 | *Enterobacter asburiae* | Secretion | 1649 | > 128 | WT | WT | N90D, N150D |
| 4098 | *Enterobacter roggenkampii* | Urine | 2457 | 4 | WT | WT | WT |
| 4162 | *Enterobacter roggenkampii* | Bronchoalveolar lavage fluid | new | > 128 | C16S | WT | N90D |
| 4121 | *Enterobacter bugandensis* | Bronchoalveolar lavage fluid | 1096 | > 128 | WT | WT | WT |
| 430 | *Enterobacter cloacae* | Bile | new | 16 | WT | WT | WT |
| 4154 | *Enterobacter ludwigii* | Secretion | 3056 | 8 | WT | WT | WT |
| 4082 | *Enterobacter mori* | Sputum | new | 32 | WT | WT | WT |

Abbreviations: MIC, the minimum inhibitory concentration of resistant subpopulations; WT, wild type; FS, frameshift mutation.

**Supplementary Table S3** The admission wards of the patients infected with 212 *Enterobacter cloacae* complex (ECC) and the NCBI accession numbers of 212 ECC strains.

| Isolates | Species | Admission wards | NCBI accession numbers |
| --- | --- | --- | --- |
| 66 | *Enterobacter hormaechei* | PICU | JAMYCZ000000000 |
| 67 | *Enterobacter hormaechei* | Pediatric department | JAMYCY000000000 |
| 68 | *Enterobacter hormaechei* | Respiratory and critical care medicine department | SAMN19790267 |
| 72 | *Enterobacter hormaechei* | Neonatal ward | [SAMN19769864](https://www.ncbi.nlm.nih.gov/biosample/SAMN19769864) |
| 73 | *Enterobacter hormaechei* | Neonatal ward | JAMYCX000000000 |
| 75 | *Enterobacter hormaechei* | Respiratory medicine department | JAMYCW000000000 |
| 76 | *Enterobacter hormaechei* | Neonatal ward | JAMYCV000000000 |
| 78 | *Enterobacter hormaechei* | Hepatobiliary surgery department | [SAMN19770947](https://www.ncbi.nlm.nih.gov/biosample/SAMN19770947) |
| 79 | *Enterobacter hormaechei* | Neonatal ward | JAMYCU000000000 |
| 112 | *Enterobacter hormaechei* | Respiratory and critical care medicine department | SAMN19789732 |
| 401 | *Enterobacter kobei* | Respiratory and critical care medicine department | JAMYDI000000000 |
| 402 | *Enterobacter asburiae* | Neonatal ward | JAMYDH000000000 |
| 403 | *Enterobacter hormaechei* | Hepatobiliary surgery department | JAMYDG000000000 |
| 404 | *Enterobacter hormaechei* | Orthopedic surgery | SAMN25233125 |
| 405 | *Enterobacter cloacae* | Medical oncologist | SAMN25233124 |
| 406 | *Enterobacter hormaechei* | Respiratory and critical care medicine department | JAMYDF000000000 |
| 408 | *Enterobacter hormaechei* | Nephropathy | JAMYDE000000000 |
| 409 | *Enterobacter hormaechei* | Infectious diseases department | JAMYDD000000000 |
| 410 | *Enterobacter hormaechei* | Gastrointestinal surgery | JAMYDC000000000 |
| 411 | *Enterobacter hormaechei* | Respiratory and critical care medicine department | SAMN25233123 |
| 412 | *Enterobacter*  *mori* | Orthopedic surgery | JAMYDB000000000 |
| 414 | *Enterobacter kobei* | Rehabilitation medicine department | SAMN25233122 |
| 415 | *Enterobacter hormaechei* | Endocrinology department | JAMYDA000000000 |
| 416 | *Enterobacter asburiae* | Respiratory medicine department | SAMN36534143 |
| 417 | *Enterobacter kobei* | Urology department | JBDYDE000000000 |
| 418 | *Enterobacter hormaechei* | Hepatobiliary surgery department | JBDYDF000000000 |
| 419 | *Enterobacter asburiae* | ICU | SAMN36534144 |
| 420 | *Enterobacter hormaechei* | Neurosurgery department | JBDYDG000000000 |
| 421 | *Enterobacter hormaechei* | Intensive care rehabilitation unit | SAMN36534145 |
| 422 | *Enterobacter roggenkampii* | Urology department | JBDYDH000000000 |
| 423 | *Enterobacter hormaechei* | Neurology department | SAMN36534146 |
| 424 | *Enterobacter hormaechei* | Gastroenterology department | JBDYDI000000000 |
| 425 | *Enterobacter hormaechei* | ICU | JBDYDJ000000000 |
| 426 | *Enterobacter hormaechei* | Burn and plastic surgery department | JBDYDK000000000 |
| 427 | *Enterobacter hormaechei* | Endocrinology department | JBDYDL000000000 |
| 428 | *Enterobacter asburiae* | Neonatal ward | JBDYDM000000000 |
| 429 | *Enterobacter hormaechei* | Burn and plastic surgery department | JBDYDN000000000 |
| 430 | *Enterobacter cloacae* | Hepatobiliary surgery department | JBDYDO000000000 |
| 4001 | *Enterobacter asburiae* | Burn and plastic surgery department | JBDYDP000000000 |
| 4002 | *Enterobacter hormaechei* | Otolaryngology head and neck surgery | JAMYDJ000000000 |
| 4003 | *Enterobacter asburiae* | Orthopedic surgery | JBDYDQ000000000 |
| 4004 | *Enterobacter roggenkampii* | Cardiology department | JBDYDR000000000 |
| 4005 | *Enterobacter hormaechei* | General Medicine department | JBDYDS000000000 |
| 4006 | *Enterobacter hormaechei* | Infectious diseases department | JBDYDT000000000 |
| 4007 | *Enterobacter hormaechei* | Neonatal ward | JBDYDU000000000 |
| 4008 | *Enterobacter hormaechei* | Urology department | JBDYDV000000000 |
| 4009 | *Enterobacter hormaechei* | NICU | JBDYDW000000000 |
| 4010 | *Enterobacter hormaechei* | Orthopedic surgery | JBDYDX000000000 |
| 4011 | *Enterobacter hormaechei* | NICU | JBDYDY000000000 |
| 4012 | *Enterobacter hormaechei* | Burn and plastic surgery department | JBDYDZ000000000 |
| 4013 | *Enterobacter hormaechei* | Medical oncologist | JBDYEA000000000 |
| 4014 | *Enterobacter ludwigii* | Hepatobiliary surgery department | JBDYEB000000000 |
| 4015 | *Enterobacter hormaechei* | ICU | JBDYEC000000000 |
| 4016 | *Enterobacter hormaechei* | Neurosurgery department | JBDYED000000000 |
| 4017 | *Enterobacter asburiae* | Neonatal ward | JBDYEE000000000 |
| 4018 | *Enterobacter roggenkampii* | Infectious diseases department | JBDYEF000000000 |
| 4019 | *Enterobacter cloacae* | Gastrointestinal surgery | JBDYEG000000000 |
| 4020 | *Enterobacter roggenkampii* | Medical oncologist | JBDYEH000000000 |
| 4021 | *Enterobacter hormaechei* | Medical oncologist | JBDYEI000000000 |
| 4023 | *Enterobacter hormaechei* | Burn and plastic surgery department | JBDYEJ000000000 |
| 4024 | *Enterobacter kobei* | Orthopedic surgery | JBDYEK000000000 |
| 4025 | *Enterobacter cloacae* | Hepatobiliary surgery department | JBDYEL000000000 |
| 4026 | *Enterobacter hormaechei* | Respiratory and critical care medicine department | JBDYEM000000000 |
| 4027 | *Enterobacter kobei* | Respiratory medicine department | JBDYEN000000000 |
| 4029 | *Enterobacter kobei* | Rehabilitation medicine department | JBDYEO000000000 |
| 4030 | *Enterobacter roggenkampii* | Geriatrics department | JBDYEP000000000 |
| 4031 | *Enterobacter hormaechei* | NICU | JBDYEQ000000000 |
| 4032 | *Enterobacter hormaechei* | Medical oncologist | JBDYER000000000 |
| 4033 | *Enterobacter hormaechei* | NICU | JBDYES000000000 |
| 4034 | *Enterobacter kobei* | Gastrointestinal surgery | JBDYET000000000 |
| 4035 | *Enterobacter hormaechei* | Hematology department | JBDYEU000000000 |
| 4036 | *Enterobacter kobei* | Gastrointestinal surgery | JBDYEV000000000 |
| 4038 | *Enterobacter cloacae* | ICU | JBDYEW000000000 |
| 4039 | *Enterobacter hormaechei* | Burn and plastic surgery department | JBDYEX000000000 |
| 4040 | *Enterobacter hormaechei* | Burn and plastic surgery department | JBDYEY000000000 |
| 4043 | *Enterobacter asburiae* | Neonatal ward | JBDYEZ000000000 |
| 4046 | *Enterobacter asburiae* | Burn and plastic surgery department | JBDYFA000000000 |
| 4047 | *Enterobacter hormaechei* | Gastrointestinal surgery | JBDYFB000000000 |
| 4048 | *Enterobacter roggenkampii* | Respiratory and critical care medicine department | JBDYFC000000000 |
| 4049 | *Enterobacter hormaechei* | Gastrointestinal surgery | JBDYFD000000000 |
| 4050 | *Enterobacter kobei* | ICU | JBDYFE000000000 |
| 4051 | *Enterobacter hormaechei* | Pediatric department | JBDYFF000000000 |
| 4053 | *Enterobacter hormaechei* | Thoracic and cardiovascular surgery department | JBDYFG000000000 |
| 4054 | *Enterobacter cloacae* | Burn and plastic surgery department | JBDYFH000000000 |
| 4055 | *Enterobacter hormaechei* | Neurosurgery department | JBDYFI000000000 |
| 4056 | *Enterobacter hormaechei* | Respiratory and critical care medicine department | JBDYFJ000000000 |
| 4058 | *Enterobacter kobei* | Urology department | JBDYFK000000000 |
| 4059 | *Enterobacter hormaechei* | Medical oncologist | JBDYFL000000000 |
| 4060 | *Enterobacter hormaechei* | Endocrinology department | JBDYFM000000000 |
| 4061 | *Enterobacter hormaechei* | Orthopedic surgery | JBDYFN000000000 |
| 4062 | *Enterobacter hormaechei* | ICU | JBDYFO000000000 |
| 4063 | *Enterobacter cloacae* | Hepatobiliary surgery department | JBDYFP000000000 |
| 4064 | *Enterobacter hormaechei* | ICU | JBDYFQ000000000 |
| 4065 | *Enterobacter hormaechei* | NICU | JBDYFR000000000 |
| 4066 | *Enterobacter asburiae* | Orthopedic surgery | JBDYFS000000000 |
| 4067 | *Enterobacter hormaechei* | Pediatric department | JBDYFT000000000 |
| 4070 | *Enterobacter kobei* | Infectious diseases department | JBDYFU000000000 |
| 4071 | *Enterobacter hormaechei* | Hepatobiliary surgery department | JBDYFV000000000 |
| 4072 | *Enterobacter cloacae* | Urology department | JBDYFW000000000 |
| 4073 | *Enterobacter hormaechei* | Neurosurgery department | JBDYFX000000000 |
| 4074 | *Enterobacter hormaechei* | Pediatric department | JBDYFY000000000 |
| 4075 | *Enterobacter kobei* | Neurosurgery department | JBDYFZ000000000 |
| 4076 | *Enterobacter mori* | Medical oncologist | JBDYGA000000000 |
| 4077 | *Enterobacter hormaechei* | Urology department | JBDYGB000000000 |
| 4079 | *Enterobacter ludwigii* | Respiratory medicine department | JBDYGC000000000 |
| 4080 | *Enterobacter hormaechei* | Breast and hernia surgery department | JBDYGD000000000 |
| 4081 | *Enterobacter bugandensis* | Respiratory and critical care medicine department | JBDYGE000000000 |
| 4082 | *Enterobacter mori* | Neonatal ward | JBDYGF000000000 |
| 4083 | *Enterobacter hormaechei* | Burn and plastic surgery department | JBDYGG000000000 |
| 4084 | *Enterobacter hormaechei* | Urology department | JBDYGH000000000 |
| 4085 | *Enterobacter hormaechei* | Medical oncologist | JBDYGI000000000 |
| 4086 | *Enterobacter hormaechei* | Cardiology department | JBDYGJ000000000 |
| 4087 | *Enterobacter hormaechei* | Hematology department | JBDYGK000000000 |
| 4088 | *Enterobacter ludwigii* | ICU | JBDYGL000000000 |
| 4089 | *Enterobacter hormaechei* | Endocrinology department | JBDYGM000000000 |
| 4090 | *Enterobacter hormaechei* | Endocrinology department | JBDYGN000000000 |
| 4091 | *Enterobacter roggenkampii* | Gastrointestinal surgery | JBDYGO000000000 |
| 4092 | *Enterobacter hormaechei* | Burn and plastic surgery department | JBDYGP000000000 |
| 4093 | *Enterobacter roggenkampii* | Burn and plastic surgery department | JBDYGQ000000000 |
| 4094 | *Enterobacter roggenkampii* | Geriatrics department | JBDYGR000000000 |
| 4095 | *Enterobacter hormaechei* | Endocrinology department | JBDYGS000000000 |
| 4096 | *Enterobacter hormaechei* | Infectious diseases department | JBDYGT000000000 |
| 4098 | *Enterobacter roggenkampii* | Neurosurgery department | JBDYGU000000000 |
| 4099 | *Enterobacter kobei* | Burn and plastic surgery department | JBDYGV000000000 |
| 4100 | *Enterobacter hormaechei* | Gastrointestinal surgery | JBDYGW000000000 |
| 4102 | *Enterobacter hormaechei* | Infectious diseases department | JBDYGX000000000 |
| 4103 | *Enterobacter roggenkampii* | NICU | JBDYGY000000000 |
| 4104 | *Enterobacter hormaechei* | Burn and plastic surgery department | JBDYGZ000000000 |
| 4105 | *Enterobacter mori* | Orthopedic surgery | JBDYHA000000000 |
| 4106 | *Enterobacter hormaechei* | ICU | JBDYHB000000000 |
| 4107 | *Enterobacter hormaechei* | Urology department | JBDYHC000000000 |
| 4108 | *Enterobacter ludwigii* | Burn and plastic surgery department | JBDYHD000000000 |
| 4109 | *Enterobacter cloacae* | Infectious diseases department | JBDYHE000000000 |
| 4110 | *Enterobacter hormaechei* | Medical oncologist | JBDYHF000000000 |
| 4111 | *Enterobacter roggenkampii* | Gastrointestinal surgery | JBDYHG000000000 |
| 4112 | *Enterobacter hormaechei* | Gastrointestinal surgery | JBDYHH000000000 |
| 4113 | *Enterobacter hormaechei* | Gastrointestinal surgery | JBDYHI000000000 |
| 4114 | *Enterobacter hormaechei* | Burn and plastic surgery department | JBDYHJ000000000 |
| 4115 | *Enterobacter asburiae* | Orthopedic surgery | JBDYHK000000000 |
| 4116 | *Enterobacter hormaechei* | Orthopedic surgery | JBDYHL000000000 |
| 4117 | *Enterobacter hormaechei* | Pediatric department | JBDYHM000000000 |
| 4118 | *Enterobacter roggenkampii* | Medical oncologist | JBDYHN000000000 |
| 4119 | *Enterobacter hormaechei* | Hepatobiliary surgery department | JBDYHO000000000 |
| 4120 | *Enterobacter hormaechei* | Neurology department | JBDYHP000000000 |
| 4121 | *Enterobacter bugandensis* | ICU | JBDYHQ000000000 |
| 4122 | *Enterobacter hormaechei* | Nephropathy | JBDYHR000000000 |
| 4123 | *Enterobacter hormaechei* | Burn and plastic surgery department | JBDYHS000000000 |
| 4124 | *Enterobacter hormaechei* | Orthopedic surgery | JBDYHT000000000 |
| 4125 | *Enterobacter hormaechei* | Hematology department | JBDYHU000000000 |
| 4127 | *Enterobacter hormaechei* | Intensive care rehabilitation unit | JBDYHV000000000 |
| 4128 | *Enterobacter hormaechei* | Burn and plastic surgery department | JBDYHW000000000 |
| 4130 | *Enterobacter hormaechei* | Orthopedic surgery | JBDYHX000000000 |
| 4131 | *Enterobacter hormaechei* | Urology department | JBDYHY000000000 |
| 4132 | *Enterobacter hormaechei* | Geriatrics department | JBDYHZ000000000 |
| 4133 | *Enterobacter hormaechei* | Hepatobiliary surgery department | JBDYIA000000000 |
| 4134 | *Enterobacter roggenkampii* | Neonatal ward | JBDYIB000000000 |
| 4135 | *Enterobacter ludwigii* | Breast and hernia surgery department | JBDYIC000000000 |
| 4136 | *Enterobacter roggenkampii* | Neonatal ward | JBDYID000000000 |
| 4137 | *Enterobacter asburiae* | ICU | JBDYIE000000000 |
| 4138 | *Enterobacter cloacae* | Burn and plastic surgery department | JBDYIF000000000 |
| 4139 | *Enterobacter hormaechei* | Burn and plastic surgery department | JBDYIG000000000 |
| 4140 | *Enterobacter bugandensis* | Pediatric department | JBDYIH000000000 |
| 4142 | *Enterobacter hormaechei* | Neurosurgery department | JBDYII000000000 |
| 4143 | *Enterobacter hormaechei* | Respiratory and critical care medicine department | JBDYIJ000000000 |
| 4144 | *Enterobacter hormaechei* | Orthopedic surgery | JBDYIK000000000 |
| 4145 | *Enterobacter kobei* | Medical oncologist | JBDYIL000000000 |
| 4146 | *Enterobacter hormaechei* | Nephropathy | JBDYIM000000000 |
| 4147 | *Enterobacter hormaechei* | Gastrointestinal surgery | JBDYIN000000000 |
| 4149 | *Enterobacter hormaechei* | Endocrinology department | JBDYIO000000000 |
| 4150 | *Enterobacter cloacae* | Neurosurgery department | JBDYIP000000000 |
| 4151 | *Enterobacter asburiae* | Pediatric department | JBDYIQ000000000 |
| 4153 | *Enterobacter hormaechei* | Burn and plastic surgery department | JBDYIR000000000 |
| 4154 | *Enterobacter ludwigii* | Burn and plastic surgery department | JBDYIS000000000 |
| 4155 | *Enterobacter hormaechei* | Pediatric department | JBDYIT000000000 |
| 4157 | *Enterobacter hormaechei* | ICU | JBDYIU000000000 |
| 4158 | *Enterobacter hormaechei* | Emergency department | JBDYIV000000000 |
| 4159 | *Enterobacter hormaechei* | Neurology department | JBDYIW000000000 |
| 4160 | *Enterobacter hormaechei* | Neurology department | JBDYIX000000000 |
| 4162 | *Enterobacter roggenkampii* | ICU | JBDYIY000000000 |
| 4163 | *Enterobacter hormaechei* | Burn and plastic surgery department | JBDYIZ000000000 |
| 4164 | *Enterobacter roggenkampii* | ICU | JBDYJA000000000 |
| 4165 | *Enterobacter kobei* | Medical oncologist | JBDYJB000000000 |
| 4166 | *Enterobacter bugandensis* | Orthopedic surgery | JBDYJC000000000 |
| 4167 | *Enterobacter hormaechei* | Orthopedic surgery | JBDYJD000000000 |
| 4168 | *Enterobacter asburiae* | ICU | JBDYJE000000000 |
| 4169 | *Enterobacter mori* | Orthopedic surgery | JBDYJF000000000 |
| 4170 | *Enterobacter hormaechei* | Hepatobiliary surgery department | JBDYJG000000000 |
| 4171 | *Enterobacter hormaechei* | Orthopedic surgery | JBDYJH000000000 |
| 4172 | *Enterobacter kobei* | NICU | JBDYJI000000000 |
| 4174 | *Enterobacter asburiae* | ICU | JBDYJJ000000000 |
| 4175 | *Enterobacter hormaechei* | Geriatrics department | JBDYJK000000000 |
| 4177 | *Enterobacter bugandensis* | Burn and plastic surgery department | JBDYJL000000000 |
| 4178 | *Enterobacter hormaechei* | Burn and plastic surgery department | JBDYJM000000000 |
| 4179 | *Enterobacter hormaechei* | Orthopedic surgery | JBDYJN000000000 |
| 4180 | *Enterobacter hormaechei* | Rehabilitation medicine department | JBDYJO000000000 |
| 4181 | *Enterobacter kobei* | Intensive care rehabilitation unit | JBDYJP000000000 |
| 4183 | *Enterobacter asburiae* | Ophthalmology department | JBDYJQ000000000 |
| 4184 | *Enterobacter hormaechei* | NICU | JBDYJR000000000 |
| 4185 | *Enterobacter hormaechei* | Hepatobiliary surgery department | JBDYJS000000000 |
| 4186 | *Enterobacter hormaechei* | Orthopedic surgery | JBDYJT000000000 |
| 4188 | *Enterobacter hormaechei* | Medical oncologist | JBDYJU000000000 |
| 4189 | *Enterobacter mori* | Geriatrics department | JBDYJV000000000 |
| 4190 | *Enterobacter hormaechei* | Burn and plastic surgery department | JBDYJW000000000 |
| 4191 | *Enterobacter hormaechei* | Respiratory and critical care medicine department | JBDYJX000000000 |
| 4192 | *Enterobacter hormaechei* | Geriatrics department | JBDYJY000000000 |
| 4193 | *Enterobacter hormaechei* | ICU | JBDYJZ000000000 |
| 4194 | *Enterobacter hormaechei* | Geriatrics department | JBDYKA000000000 |
| 4195 | *Enterobacter cloacae* | Hepatobiliary surgery department | JBDYKB000000000 |
| 4196 | *Enterobacter hormaechei* | Endocrinology department | JBDYKC000000000 |
| 4197 | *Enterobacter asburiae* | Neurology department | JBDYKD000000000 |
| 4199 | *Enterobacter hormaechei* | ICU | JBDYKE000000000 |
| 4200 | *Enterobacter hormaechei* | Pediatric department | JBDYKF000000000 |

The ST177 strains were highlighted in red.

Abbreviations: NCBI, national center for biotechnology information; PICU, pediatric intensive care unit; ICU, intensive care unit; NICU, neonatal intensive care unit.

**Supplementary Table S4** Colistin MICs in the absence and presence of efflux pump inhibitors in 26 colistin-heteroresistant parental strains and resistant subpopulations.

|  | | | | | | Parental strains | | | | | | |  | | | | | | | Resistance subpopulations | | | | | |  |
| --- | --- | --- | --- | --- | --- | --- | --- | --- | --- | --- | --- | --- | --- | --- | --- | --- | --- | --- | --- | --- | --- | --- | --- | --- | --- | --- |
| Isolates | COL^a^ | | COL +  CCCP^b^ | | COL +  PAβN^b^ | | | Fold change  with CCCP^c^ | | Fold change  With PAβN^d^ | |  | | | COL^a^ | | COL +  CCCP^b^ | | COL +  PAβN^b^ | | | Fold change  with CCCP^c^ | | Fold change  With PAβN^d^ | |  |
| 68 | | 0.5 | | 0.5 | | | < 0.125 | | 1 | | **> 4** | | |  | | 8 | | 1 | | | 8 | | **8** | | 1 | |
| 79 | | 2 | | 1 | | | < 0.125 | | 2 | | **> 16** | | |  | | 16 | | 1 | | | 16 | | **16** | | 1 | |
| 418 | | 0.5 | | 0.5 | | | < 0.125 | | 1 | | **> 4** | | |  | | 8 | | 1 | | | 8 | | **8** | | 1 | |
| 428 | | 2 | | 2 | | | 2 | | 1 | | 1 | | |  | | > 128 | | 2 | | | > 128 | | **> 64** | | 1 | |
| 430 | | 0.5 | | 0.25 | | | 0.5 | | 2 | | 1 | | |  | | 16 | | 1 | | | 16 | | **16** | | 1 | |
| 4002 | | 0.5 | | 0.5 | | | < 0.125 | | 1 | | **> 4** | | |  | | 4 | | 0.25 | | | 0.25 | | **16** | | **16** | |
| 4012 | | 2 | | 0.5 | | | 0.25 | | **4** | | **8** | | |  | | 8 | | 0.25 | | | 0.25 | | **32** | | **32** | |
| 4027 | | NA^e^ | | ND^f^ | | | ND^f^ | | ND^f^ | | ND^f^ | | |  | | 128 | | 1 | | | 4 | | **128** | | **32** | |
| 4029 | | NA^e^ | | ND^f^ | | | ND^f^ | | ND^f^ | | ND^f^ | | |  | | > 128 | | 0.5 | | | 128 | | **> 256** | | 1 | |
| 4035 | | 1 | | 0.5 | | | < 0.125 | | 2 | | **> 8** | | |  | | 16 | | 1 | | | 16 | | **16** | | 1 | |
| 4046 | | NA | | ND^f^ | | | ND^f^ | | ND^f^ | | ND^f^ | | |  | | > 128 | | 0.5 | | | 1 | | **> 256** | | **> 128** | |
| 4049 | | 2 | | 0.5 | | | 2 | | **4** | | 1 | | |  | | 8 | | 1 | | | 8 | | **8** | | 1 | |
| 4058 | | 2 | | 1 | | | 2 | | 2 | | 1 | | |  | | 128 | | 1 | | | 128 | | **128** | | 1 | |
| 4067 | | 2 | | 1 | | | 2 | | 2 | | 1 | | |  | | 8 | | 1 | | | 8 | | **8** | | 1 | |
| 4075 | | NA^e^ | | ND^f^ | | | ND^f^ | | ND^f^ | | ND^f^ | | |  | | > 128 | | 1 | | | 2 | | **> 128** | | **> 64** | |
| 4082 | | 2 | | 0.5 | | | 2 | | **4** | | 1 | | |  | | 32 | | 0.5 | | | 32 | | **64** | | 1 | |
| 4083 | | 1 | | 1 | | | 1 | | 1 | | 1 | | |  | | 8 | | 1 | | | 4 | | **8** | | 2 | |
| 4089 | | 2 | | 1 | | | 0.25 | | 2 | | **8** | | |  | | 16 | | < 0.125 | | | < 0.125 | | **> 128** | | **> 128** | |
| 4121 | | 2 | | 1 | | | 2 | | 2 | | 1 | | |  | | > 128 | | 0.5 | | | 128 | | **> 256** | | 1 | |
| 4145 | | NA^e^ | | ND^f^ | | | ND^f^ | | ND^f^ | | ND^f^ | | |  | | > 128 | | 0.5 | | | 0.25 | | **> 256** | | **> 512** | |
| 4149 | | 0.5 | | 0.5 | | | 0.25 | | 1 | | 2 | | |  | | 8 | | 0.5 | | | 0.25 | | **16** | | **32** | |
| 4154 | | 1 | | 0.5 | | | 0.25 | | 2 | | **4** | | |  | | 8 | | 0.5 | | | 0.25 | | **16** | | **32** | |
| 4158 | | 2 | | 0.5 | | | 0.5 | | **4** | | **4** | | |  | | 4 | | 0.5 | | | 0.5 | | **8** | | **8** | |
| 4162 | | NA^e^ | | ND^f^ | | | ND^f^ | | ND^f^ | | ND^f^ | | |  | | > 128 | | < 0.125 | | | < 0.125 | | **> 1024** | | **> 1024** | |
| 4190 | | 2 | | < 0.125 | | | < 0.125 | | **> 16** | | **> 16** | | |  | | 8 | | < 0.125 | | | < 0.125 | | **> 64** | | **> 64** | |
| 4192 | | 2 | | 0.5 | | | 2 | | **4** | | 1 | | |  | | 8 | | 1 | | | 8 | | **8** | | 1 | |
| ATCC 25922 | | 1 | | 0.25 | | | 1 | | **4** | | 1 | | |  | | ND^f^ | | ND^f^ | | | ND^f^ | | ND^f^ | | ND^f^ | |

^a^COL, colistin MIC (mg/L).

^b^COL + CCCP, colistin MIC (mg/L) in the presence of carbonyl cyanide 3-chlorophenylhydrazone; colistin MIC (mg/L) in the presence of Phenyl-Arginine β-Naphthylamide.

^c^Fold change with CCCP, colistin MIC decreased fold change under the pressure of CCCP (10mg/L).

^d^Fold change With PAβN, colistin MIC decreased fold change under the pressure of PAβN (25mg/L).

^e^NA, the results were not visible due to the occurrence of the skip well phenomenon in multiple tests, rendering the results unexplainable.

^f^ND, the MIC was not detected.

Bold indicated that the efflux pump inhibitors reduced the MIC of the isolates by at least fourfold.
