# Supplementary material for: Clinical characteristics, molecular epidemiology and mechanisms of colistin heteroresistance in Enterobacter cloacae complex
Source: Front Cell Infect Microbiol. 2025 Mar 6;15:1536058. doi: 10.3389/fcimb.2025.1536058 (PMC11922889; doi:10.3389/fcimb.2025.1536058)
Supplement: Supplementary file 2 [file DataSheet1.docx]

**
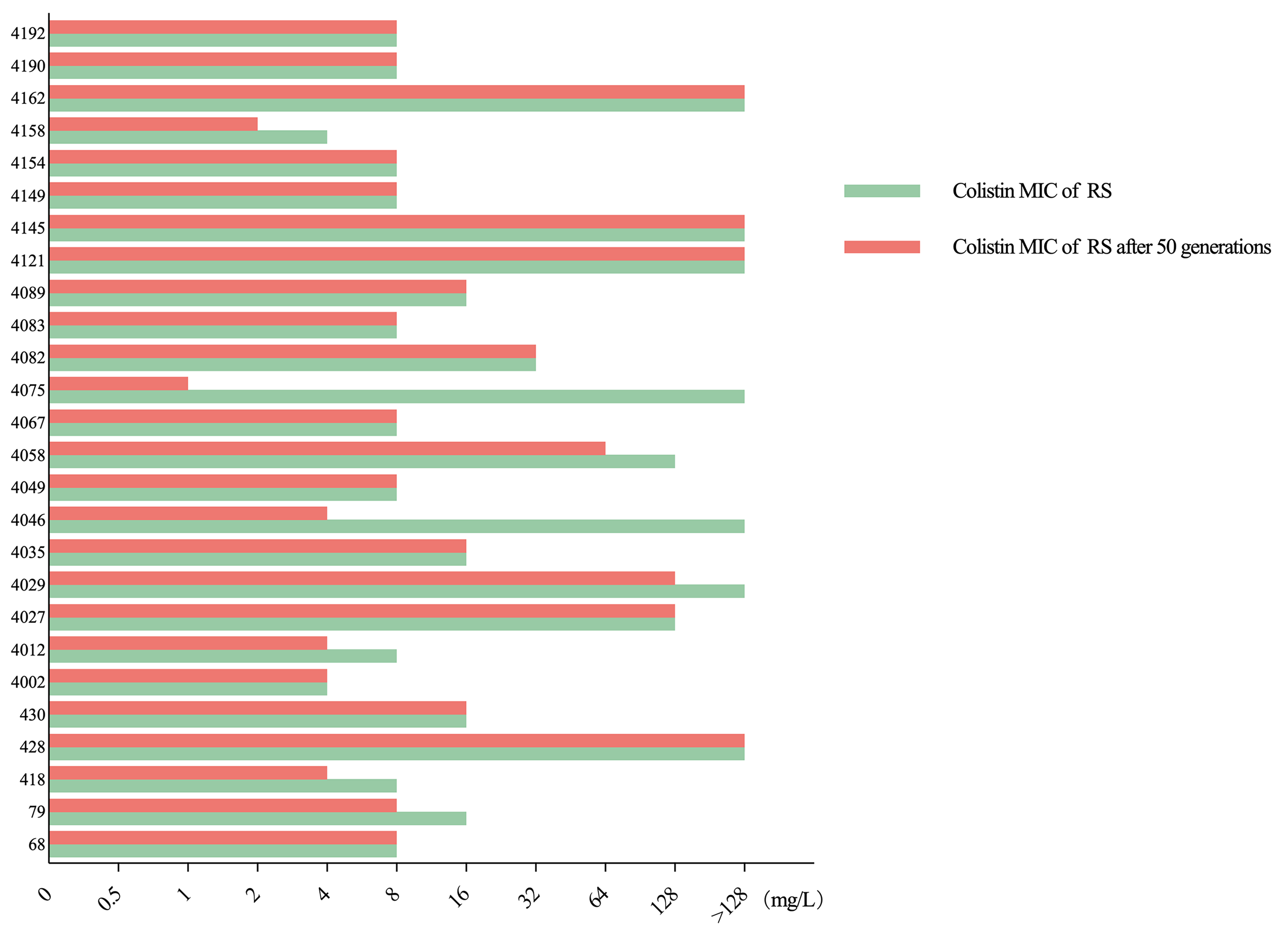
**

**Supplementary Figure S1** The minimum inhibitory concentrations (MICs) of resistant subpopulations collected from the highest colistin concentration plates in the population analysis profiles (PAP) analysis were determined after 50 generations in Mueller-Hinton broth. The aim was to assess the stability of colistin-heteroresistant isolates.

**
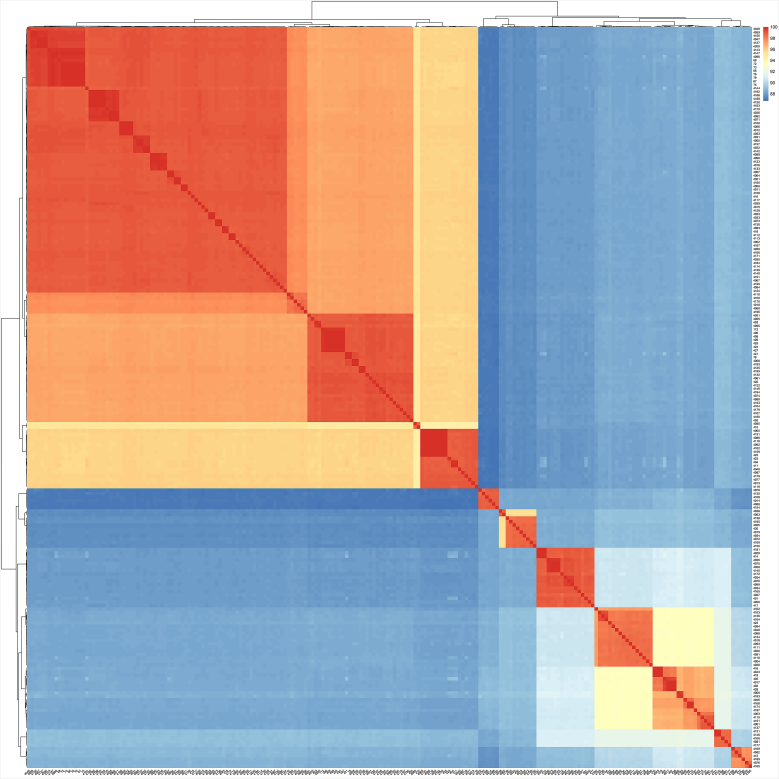
**

**Supplementary Figure S2** Heat map based on average nucleotide identity (ANI) values between each pair of genome sequences from 212 ECC strains.
